# Volatile composition of essential oils from major Egyptian *Citrus* cultivars: a multivariate chemometric approach

**DOI:** 10.1038/s41598-026-60409-w

**Published:** 2026-07-06

**Authors:** Abeer H. Elmaidomy, Esraa M. Mohamed, Hebatallah S. Bahr, Usama Ramadan Abdelmohsen

**Affiliations:** 1 Department of Pharmacognosy, Faculty of Pharmacy, Beni-Suef University, Beni-Suef, 62514 Egypt; 2 Department of Pharmacognosy, Faculty of Pharmacy, MUST, Giza, 12566 Egypt; 3 Department of Pharmacognosy, Faculty of Pharmacy, Nahda University, Beni-Suef, 62513 Egypt; 4 Department of Pharmacognosy, Faculty of Pharmacy, Deraya University, Minia, 61111 Egypt; 5 Department of Pharmacognosy, Faculty of Pharmacy, Minia University, Minia, Egypt

**Keywords:** *Citrus* species, Volatile organic compounds, PCA, PLS-DA, sPLS-DA, Biochemistry, Plant sciences

## Abstract

The essential oils extracted from the rind of *Citrus* spp. (F. Rutaceae) is abundant in chemical components of importance for the food and perfume industries, and therefore has been thoroughly researched during the past few decades. In this manuscript, we provide a comprehensive study of the volatile composition of this oils for the 12 most cultivated *Citrus* in Egypt: *Citrus sinensis* varieties: “Washington Navel (Navelina)”, “Balady Seeded Orange”, “Blood Orange”, and “Sweet Orange Sukkari”, *Citrus reticulata* Blanco varieties: “Yousfy Balady”, “Ponkan Chinese Honey Mandarin”, “Murcott Mandarin”, “Clementine Mandarin”, and “Dancy Tangerine”, *Citrus aurantifolia* Swingle: “Key Lime”, *Citrus limettioides*: “Sweet Lime”, and *Citrus paradisi* Macf.: “Tangelo Seminole”. All 12 varieties have been shown to contain 49 volatile chemical compounds, the majority of which are terpenoid (90–100%). However, some of the volatile compounds found in *Citrus* peel are non-terpenoid, and some of them may be helpful as species biomarkers. Multivariate data analysis was applied including unsupervised (PCA) and supervised multivariate approaches (PLS-DA, sPLS-DA) to profile volatile compounds across three *Citrus* groups (*C. reticulate* varieties, *C. sinensis* varieties, and other *Citrus* species). PCA revealed partial overlap between *C. reticulata* and *C. sinensis*, while *C. paradisi* and *C. aurantifolia* were clearly separated. PLS-DA and sPLS-DA confirmed these distinctions, with sPLS-DA achieving stronger discrimination. Variable Importance in Projection (VIP) analysis identified key discriminant metabolites, including α-ocimene, neryl acetate, and germacrene B, as primary contributors to varietal separation. These findings reveal distinct volatile profiles among Citrus groups and demonstrate the applicability of multivariate analysis for Citrus differentiation.

**Supplementary Information:**

The online version contains supplementary material available at 10.1038/s41598-026-60409-w.

## Introduction

The genus *Citrus* (family Rutaceae, subfamily Aurantioideae) comprises several economically important fruit species, including sweet orange (*Citrus* sinensis (L.) Osbeck), mandarin (*Citrus* reticulata Blanco), *Citrus paradisi* Macf.: “Tangelo Seminole”, *Citrus aurantifolia* Swingle: “Key Lime”, and sour orange (*Citrus* aurantium L.)^1,2^. *Citrus* species are predominantly diploid and are believed to have originated in tropical and subtropical regions of Southeast Asia. Owing to their remarkable adaptability and high economic value, *Citrus* crops are now cultivated extensively across tropical, subtropical, arid, and semi-arid regions worldwide, making them among the most important fruit crops in global horticulture^3^.

*Citrus* fruits play a pivotal role in international agricultural trade and human nutrition due to their high content of vitamins, minerals, dietary fiber, and bioactive phytochemicals. Their widespread consumption and diverse industrial applications have contributed significantly to the expansion of *Citrus* production worldwide^3^. In Egypt, *Citrus* cultivation has been practiced since ancient times and has developed into a major horticultural industry. Several commercially important cultivars are cultivated across different agroecological regions, including Balady orange, Sukkari sweet orange, blood orange, mandarin, lemon, and grapefruit. Recent agricultural statistics indicate that *Citrus* occupies approximately 204,095 ha, representing nearly 29% of the total fruit-growing area in Egypt (700,854 ha). The productive area exceeds 175,734 ha and yields approximately 4.27 million metric tons annually, of which nearly 1.34 million metric tons are exported^4^. Consequently, Egypt ranks among the leading *Citrus*-producing countries worldwide and is one of the largest exporters of fresh *Citrus* fruits, with *Citrus* representing the country’s most valuable horticultural export commodity and a major source of foreign currency earnings^5^.

Beyond their importance as fresh fruits, *Citrus* species constitute one of the most important natural sources of essential oils (EOs), which are widely utilized in the food, pharmaceutical, nutraceutical, and cosmetic industries. *Citrus* essential oils are predominantly extracted from the fruit peel (flavedo), although flowers and leaves are also valuable sources of volatile constituents. These oils are highly valued for their characteristic aromas, flavoring properties, and diverse biological activities, including antioxidant, antimicrobial, anti-inflammatory, and insecticidal effects^6–9^. The volatile and semi-volatile fraction typically accounts for 85–99% of the total oil composition and may contain more than 200 identified compounds^6,9^. Monoterpene and sesquiterpene hydrocarbons generally dominate the volatile profile, accompanied by oxygenated derivatives such as alcohols, aldehydes, ketones, esters, and acids. In contrast, the non-volatile fraction mainly consists of flavonoids, coumarins, sterols, diterpenoids, and fatty acids^10^. Because the composition of *Citrus* essential oils varies among species, cultivars, and growing environments, volatile metabolite profiling has become an important tool for assessing *Citrus* diversity, authenticity, and quality.

Recent advances in metabolomics have greatly enhanced the characterization of plant volatile organic compounds (VOCs), enabling comprehensive evaluation of metabolic variation among closely related taxa. In particular, gas chromatography-based metabolomic approaches coupled with multivariate statistical analyses provide powerful tools for investigating complex phytochemical datasets. Chemometric techniques such as Principal Component Analysis (PCA), Partial Least Squares Discriminant Analysis (PLS-DA), and sparse Partial Least Squares Discriminant Analysis (sPLS-DA) facilitate the identification of discriminant metabolites, reveal patterns of chemical diversity, and support classification and authentication of plant materials^11–13^. These approaches have been successfully applied to differentiate *Citrus* species and cultivars based on their volatile signatures and to identify potential chemical markers associated with botanical origin and product quality.

Despite the economic importance of *Citrus* production in Egypt, comprehensive comparative studies addressing the volatile composition of the major cultivated *Citrus* varieties remain limited. Therefore, the present study aimed to investigate the volatile profiles of peel essential oils from the twelve most widely cultivated *Citrus* cultivars in Egypt, including *Citrus sinensis* varieties: “Washington Navel (Navelina)”, “Balady Seeded Orange”, “Blood Orange”, and “Sweet Orange Sukkari”, *Citrus reticulata* Blanco varieties: “Yousfy Balady”, “Ponkan Chinese Honey Mandarin”, “Murcott Mandarin”, “Clementine Mandarin”, and “Dancy Tangerine”, *Citrus aurantifolia* Swingle: “Key Lime”, *Citrus limettioides*: “Sweet Lime”, and *Citrus paradisi* Macf.: “Tangelo Seminole”. Furthermore, a metabolomics-driven chemometric strategy incorporating PCA, PLS-DA, and sPLS-DA was employed to evaluate metabolic variability among *Citrus* groups, establish robust classification models, and identify key discriminant VOCs that may serve as biomarkers for *Citrus* authentication, quality assessment, and industrial standardization.

## Materials and methods

### Fruits collection

The full mature fruits of *Citrus sinensis* varieties: “Washington Navel (Navelina)”, “Balady Seeded Orange”, “Blood Orange”, and “Sweet Orange Sukkari”, *Citrus reticulata* Blanco varieties: “Yousfy Balady”, “Ponkan Chinese Honey Mandarin”, “Murcott Mandarin”, “Clementine Mandarin”, and “Dancy Tangerine”, *Citrus aurantifolia* Swingle: “Key Lime”, *Citrus limettioides*: “Sweet Lime”, and *Citrus paradisi* Macf.: “Tangelo Seminole”, were collected in September 2022 to February 2023, included in this study were collected from a private house garden located on Atia Street, Beni-Suef, Egypt (Table 1). Dr. Abd El-Halim A. Mohammed of the Horticulture Research Institute’s Department of Flora and Phytotaxonomy Research in Dokki, Cairo, Egypt; graciously provided the identifications. Voucher herbarium specimens, designated as accession numbers 2021-BuPD-100–101 and 2021-BuPD-105–114, were deposited in the Herbarium of the Department of Pharmacognosy, Faculty of Pharmacy, Beni-Suef University, Egypt. Following harvest, *Citrus* fruits were transported to the laboratory, where they were sorted to ensure uniformity in size, color, and maturity and to exclude damaged or diseased fruits. The selected fruits were washed, dried with towel, labeled, and stored under 4^◦^C until analysis.

**Table 1:** Environmental and cultivation conditions in different *Citrus* varieties from Egypt.

Number	Citrus variety	Environmental and cultivation conditions
1.	Citrus sinensis variety: “Washington Navel (Navelina)”	Geographical location: 29.08318250172236, 31.094391431381126. Irrigation cycle: 1 to 2 times per week depending on rainfall in the area. Ripening stage: Full mature fruits. Harvesting Time: 21 January 2023, at 7:00 AM, near 18° C. Part of the plant used: Peels.
2.	*Citrus sinensis* variety: “Balady Seeded Orange”	Geographical location: 29.08318250172236, 31.094391431381126. Irrigation cycle: 1 to 2 times per week depending on rainfall in the area. Ripening stage: Full mature fruits. Harvesting Time: 21 December 2022 at 7:00 AM, near 21 °C. Part of the plant used: Peels.
3.	*Citrus sinensis* variety: “Blood Orange”	Geographical location: 29.08318250172236, 31.094391431381126. Irrigation cycle: 1 to 2 times per week depending on rainfall in the area. Ripening stage: Full mature fruits. Harvesting Time: 21 January 2023, at 7:00 AM, near 18° C. Part of the plant used: Peels.
4.	*Citrus sinensis* variety: “Sweet Orange Sukkari”	Geographical location: 29.08318250172236, 31.094391431381126. Irrigation cycle: 1 to 2 times per week depending on rainfall in the area. Ripening stage: Full mature fruits. Harvesting Time: 21 January 2023, at 7:00 AM, near 18° C. Part of the plant used: Peels.
5.	*Citrus reticulata* Blanco variety: “Yousfy Balady”	Geographical location: 29.08318250172236, 31.094391431381126. Irrigation cycle: 1 to 2 times per week depending on rainfall in the area. Ripening stage: Full mature fruits. Harvesting Time: 21 December 2022 at 7:00 AM, near 21 °C. Part of the plant used: Peels.
6.	*Citrus reticulata* Blanco variety: “Ponkan Chinese Honey Mandarin”	Geographical location: 29.08318250172236, 31.094391431381126. Irrigation cycle: 1 to 2 times per week depending on rainfall in the area. Ripening stage: Full mature fruits. Harvesting Time: 21 December 2022 at 7:00 AM, near 21 °C. Part of the plant used: Peels.
7.	*Citrus reticulata* Blanco variety: “Murcott Mandarin”	Geographical location: 29.08318250172236, 31.094391431381126. Irrigation cycle: 3 days per week depending on rainfall in the area. Ripening stage: Full mature fruits. Harvesting Time: 21 February 2023 at 7:00 AM, near 18° C. Part of the plant used: Peels.
8.	*Citrus reticulata* Blanco variety: “Clementine Mandarin”	Geographical location: 29.08318250172236, 31.094391431381126. Irrigation cycle: 1 to 2 times per week depending on rainfall in the area. Ripening stage: Full mature fruits. Harvesting Time: 21 December 2022 at 7:00 AM, near 21 °C. Part of the plant used: Peels.
9.	*Citrus reticulata* Blanco variety: “Dancy Tangerine”	Geographical location: 29.08318250172236, 31.094391431381126. Irrigation cycle: 1 to 2 times per week depending on rainfall in the area. Ripening stage: Full mature fruits. Harvesting Time: 21 January 2023, at 7:00 AM, near 18° C. Part of the plant used: Peels.
10.	*Citrus aurantifolia* Swingle: “Key Lime”	Geographical location: 29.08318250172236, 31.094391431381126. Irrigation cycle: 2 to 3 times per week, avoiding over-saturation to prevent fruit splitting or root rot. Ripening stage: Full mature fruits. Harvesting Time: 1 September 2022 at 7:00 AM, near 27° C. Part of the plant used: Peels.
11.	*Citrus limettioides*: “Sweet Lime”	Geographical location: 29.08318250172236, 31.094391431381126. Irrigation cycle: 3 days per week depending on rainfall in the area. Ripening stage: Full mature fruits. Harvesting Time: 21 February 2023 at 7:00 AM, near 18° C. Part of the plant used: Peels.
12.	*Citrus paradisi* Macf.: “Tangelo Seminole”	Geographical location: 29.08318250172236, 31.094391431381126. Irrigation cycle: 1 to 2 times per week depending on rainfall in the area. Ripening stage: Full mature fruits. Harvesting Time: 21 January 2023, at 7:00 AM, near 18° C. Part of the plant used: Peels.

### Sample preparation

The fresh peels (0.25 kg) were ground and hydrodistilled separately for two hours at 75 °C using the Clevenger apparatus. The oils were collected individually, dried over anhydrous sodium sulphate, and stored at 4 °C in sealed amber glass vials. The yield (v/w%) was calculated based on the fresh weight of the plant material^14–16^.

### GC-MS analysis

The oil extracted from peels was subjected to separate chromatographic examination using gas chromatography-mass spectrometry (GC/MS)^14–17^. The GC-MS device combines a TRACE GC ultra-high performance gas chromatograph (THERMO Scientific Corp., USA) with a thermal mass spectrometer detector (ISQ single quadrupole mass spectrometry). The GC-MS system was equipped with a TR-5 MS column (30 m x 0.32 mm i.d., 0.25 mm film thickness). Helium served as the carrier gas for the study, and the following temperature program was utilized to set the split ratio at 1:10: 60 C for a minute, then 4.0 C/min to 240 C, with a hold of one minute. Both the injector and the detector were kept at 210 °C. The mixes were consistently given in one-liter diluted samples (1:10 hexane, v/v). Mass spectra were generated utilizing a spectral range of m/z 40–450 and electron ionization (EI) at 70 eV. The chemical components of the essential oil were deconvoluted and identified using AMDIS software (www.amdis.net) based on their retention indices (in relation to n-alkanes C8–C22), mass spectra that matched authentic standards, and retention periods (where available). (78 Version 5.10 of the NIST Standard Reference Database) Collection of Wiley Spectral Library^14–17^.

### Multivariate and statistical analysis

Data were prepared as described by^11,12,13^ and uploaded to the MetaboAnalyst 6.0 web server (https://www.metaboanalyst.ca ). The data matrix consisted of the relative peak area percentages of the identified volatile compounds obtained by GC-MS analysis. Samples were classified into three citrus groups (*Citrus sinensis*, *Citrus reticulata*, and other citrus species). Data preprocessing included normalization by sum, logarithmic transformation, and auto-scaling (mean-centered and divided by the standard deviation). Both univariate and multivariate analyses were performed. One-way analysis of variance (ANOVA) was used. Unsupervised principal component analysis (PCA) was applied to explore intrinsic clustering patterns, whereas supervised partial least squares-discriminant analysis (PLS-DA) and sparse partial least squares-discriminant analysis (sPLS-DA) were employed. Variable Importance in Projection (VIP) scores were calculated. Hierarchical clustering analysis and K-means clustering were performed using Euclidean distance and Ward’s linkage method. Graphs and visualizations were generated using the MetaboAnalyst^®^ platform.

## Results

Studies indicate that the chemical composition of essential oils is influenced by factors such as plant age, harvest timing, geographical origin, environmental conditions, and the methods used for drying and extraction^18^. Moreover, in the present study, the oils were subjected to the high temperatures of the distillation process, which can trigger a range of acid-catalyzed reactions that have been widely documented in the literature^18^. These reactions mainly involve the rearrangement of *α*- and *β*-pinene, α-phellandrene, sabinene, and α-thujene, resulting in the formation of compounds such as α-terpineol, terpinen-4-ol, borneol, terpinolene, limonene, and γ- and α-terpinene. In contrast, citral (neral and geranial) is thought to undergo polymerization during distillation, while the hydrolysis of geranyl and neryl acetates is considered responsible for the increased levels of α-terpineol^18,19^, (Tables 2, 3, 4 and 5). Additionally, some authors have reported that essential oils produced by hydrodistillation contain higher levels of oxygenated terpene compounds than those obtained using the cold-press method^18,20,21^.

### GC-MS profiling of Citrus sinensis varieties: “Washington Navel (Navelina)”, “Balady Seeded Orange”, “Blood Orange”, and “Sweet Orange Sukkari” peel oils

The peels of Egyptian *C. sinensis* variety: “Washington Navel (Navelina)” oranges yielded 2.00% (v/w) volatile oil on a fresh weight basis. The oil was colorless, exhibited a characteristic aroma, was lighter than water, and appeared clear, transparent, and non-viscous at both room temperature and 4 °C. GC–MS analysis identified a total of 17 constituents, representing 100.00% of the detected compounds. (Table 2, Figures S1A, 2). The identified constituents (**1–17**) were classified into different chemical groups, mainly monoterpenes and sesquiterpenes (Table 2). Monoterpenes constituted the predominant fraction of the oil (97.65%), while sesquiterpenes accounted for only 2.35% of the total identified components (Table 2). Fourteen monoterpene compounds (97.65%) were detected, ranging from cyclic hydrocarbons—such as D-limonene (**8**), γ-terpinene (**11**), and p-mentha-1,4(8)-diene (**12**), which together represented the major portion of the oil (68.22%)—to oxygenated acyclic compounds, including acetonyldimethylcarbinol (**1**), 2-hexenal (**2**), linalool (**15**), (R)-(+)-citronellal (**16**), decanal (**19**), and α-citral (**20**) (4.03%). Acyclic hydrocarbons, represented mainly by α-myrcene (**6**), contributed 13.72%, while bicyclic hydrocarbons—such as 2-thujene (**3**), α-pinene (**4**), (+)-sabinene (**5**), and 3-carene (**9**)—accounted for 11.68% (Table 2, Figure S2). In addition, three bicyclic sesquiterpene hydrocarbons were identified, namely caryophyllene (**23**), eremophila-1 (**10**),11-diene (**24**), and α-cadinene5 (**25**), collectively representing 2.35% of the oil (Table 2; Figures S1A and 2).

Egyptian *C. sinensis* variety: “Balady Seeded Orange” peels yielded 3.0% (v/w) volatile oil on a fresh weight basis. The oil was colorless, possessed a characteristic aroma, was lighter than water, and appeared clear, transparent, and non-viscous at both room temperature and 4 °C. GC–MS analysis allowed the identification of 19 constituents, representing 99.98% of the total detected compounds (Table 2; Figures S1B and 2). The identified components (**1–19**) belonged mainly to the monoterpene and sesquiterpene classes (Table 2). Monoterpenes constituted the major fraction (97.70%), whereas sesquiterpenes accounted for 2.28% of the total oil composition (Table 2). Seventeen monoterpene compounds (97.70%) were detected, ranging from cyclic hydrocarbons—such as α-phellandrene (**7**), D-limonene (**8**), and p-mentha-1,4(8)-diene (**12**), which together formed the principal oil fraction (62.59%)—to oxygenated cyclic hydrocarbons, exemplified by terpinen-4-ol (17, 0.27%). Oxygenated acyclic compounds, including nonanal (**13**), 1-octanol (**14**), linalool (**15**), (R)-(+)-citronellal (**16**), decanal (**19**), α-citral (**20**), and lauraldehyde (**22**), contributed 10.90%, while acyclic hydrocarbons, mainly α-myrcene (**6**), accounted for 14.82%. Bicyclic hydrocarbons—α-pinene (**4**), (+)-sabinene (**5**), 3-carene (**9**), and camphene (**10**)—represented 8.26%, and the oxygenated bicyclic hydrocarbon (–) -(1 S,2R,4R)-β-fenchol (**18**) was present at 0.86% (Table 2). In addition, two bicyclic sesquiterpene hydrocarbons, eremophila-1(**10**),11-diene (**24**) and α-cadinene (**25**), were identified, together comprising 2.28% of the total oil composition (Table 2; Figures S1B and 2).

Egyptian *C. sinensis* variety: “Blood Orange” peels yielded 2.5% (v/w) volatile oil based on fresh weight. The obtained oil was colorless, possessed a characteristic aroma, was lighter than water, and appeared clear, transparent, and non-viscous at both room temperature and 4 °C. GC–MS analysis led to the identification of 15 constituents, accounting for 100.00% of the total detected compounds (Table 2; Figures S1C and 2).

The identified components (1–15) were distributed among different chemical classes, mainly monoterpenes and sesquiterpenes (Table 2). Monoterpenes dominated the oil composition, representing 99.06% of the total constituents, whereas sesquiterpenes constituted only 0.94% (Table 2). Twelve monoterpene compounds (99.06%) were detected, ranging from cyclic hydrocarbons—such as D-limonene (8) and p-mentha-1,4(8)-diene (12), which together formed the major fraction of the oil (64.27%)—to oxygenated acyclic compounds, including linalool (15), (R)-(+)-citronellal (16), decanal (19), and α-citral (20), accounting for 8.26%. Acyclic hydrocarbons, represented mainly by α-myrcene (6), contributed 14.29%, while bicyclic hydrocarbons—2-thujene (3), α-pinene (4), (+)-sabinene (5), and 3-carene (9)—accounted for 11.60%. The oxygenated bicyclic hydrocarbon (–) -(1 S,2R,4R)-β-fenchol (18) was present at 0.64% (Table 2). Additionally, three sesquiterpene constituents (0.94%) were identified, including bicyclic hydrocarbons (eremophila-1(10),11-diene (24) and α-cadinene (25)) and a tricyclic hydrocarbon (α-copaene (21)) (Table 2; Figures S1C and 2).

Egyptian *C. sinensis* variety: “Sweet Orange Sukkari” peels produced 1.80% (v/w) volatile oil on a fresh weight basis. The oil was colorless, exhibited a characteristic aroma, was lighter than water, and appeared clear, transparent, and non-viscous at both room temperature and 4 °C. GC–MS analysis identified four constituents, which together accounted for 100.00% of the detected compounds (Table 2; Figures S1D and 2). All identified constituents (1–5) belonged exclusively to the monoterpene class (Table 2), representing 100.00% of the oil composition. These monoterpenes ranged from cyclic hydrocarbons, with D-limonene (8) as the dominant component (91.23%), to oxygenated acyclic compounds such as linalool (15, 1.04%), acyclic hydrocarbons represented by α-myrcene (6, 4.59%), and bicyclic hydrocarbons exemplified by α-pinene (4, 3.14%) (Table 2).

In the present study, the essential oils obtained from fully mature peels of *C. sinensis* varieties—Washington navel (Navelina), balady seeded orange, blood orange, and sweet orange (Sukkari)—were characterized by a predominance of monoterpenes, accounting for 97.5–100% of the total oil composition (Table 2). Sweet orange (Sukkari) oil consisted exclusively of monoterpene constituents (100.00%), whereas the oils from Washington navel (Navelina), balady seeded orange, and blood orange contained slightly lower monoterpene levels (97.65%, 97.70%, and 99.06%, respectively). Noticeable quantitative differences were observed among the analyzed *Citrus* essential oils. In particular, limonene abundance decreased in the following order: sweet orange (Sukkari) > Washington navel (Navelina) > blood orange > balady seeded orange. Significant variations were also detected in the levels of 2-thujene, α-pinene, (+)-sabinene, (+)-β-pinene, and α-myrcene (Table 2). Moreover, balady seeded orange oil was distinguished by a relatively higher proportion of oxygenated terpenes (12.03% of the total oil) compared with Washington navel (Navelina), blood orange, and sweet orange (Sukkari) oils (4.03%, 8.90%, and 1.04%, respectively) (Table 2). Conversely, the essential oils of all *C. sinensis* cultivars examined were characterized by a low sesquiterpene content, not exceeding 2.50% (Table 2).

Previous reports indicate notable geographical variations in the chemical composition of *C. sinensis* peel essential oils. Nigerian samples differed from Egyptian ones; for instance, peels collected from Ota were rich in limonene (59.3%), terpineol (8.31%), linalool (6.56%), and citronellol (6.21%)^22^. In contrast, *C. sinensis* cultivated in North-East Egypt was reported to contain 13 constituents, representing about 99.77% of the total oil, with D-limonene, terpinene, and myrcene as the predominant components^23^. Essential oils from Pakistani *C. sinensis* peels were found to comprise 18 constituents (100.00%), with limonene as the dominant compound (80.90%), followed by myrcene (4.19%)^24^. Similarly, Kenyan *C. sinensis* peel oils were characterized by a high proportion of monoterpenes and a relatively low sesquiterpene content, with limonene, α-pinene, sabinene, and α-terpinene identified as the major constituents^25^. The peel essential oil of Bingtang sweet orange was also investigated, revealing that monoterpene and sesquiterpene hydrocarbons constituted 96.03% (w/w) of the oil. Limonene was the principal component (77.49%), followed by myrcene (6.27%), α-farnesene (3.64%), γ-terpinene (3.34%), α-pinene (1.49%), sabinene (1.29%), along with several minor compounds^26^. Furthermore, analysis of cold-pressed Gannan navel orange peel oil and its molecular distillation light fraction identified 27 and 20 constituents, respectively. In both cases, limonene predominated, accounting for 85.32% of the cold-pressed oil and 60.44% of the light phase fraction^27^.

### GC-MS profiling of *Citrus reticulata* Blanco varieties: “Yousfy Balady”, “Ponkan Chinese Honey Mandarin”, “Murcott Mandarin”, “Clementine Mandarin”, and “Dancy Tangerine” peel oils

Egyptian *C. reticulata* Blanco variety: “Yousfy Balady” peels yielded 1.50% (v/w) volatile oil based on fresh weight. The oil was colorless, exhibited a characteristic aroma, was lighter than water, clear, and transparent, and displayed blue fluorescence due to the presence of anthranilic acid, N-methyl-, methyl ester (1.67%) (Table 3). It was non-viscous at both room temperature and 4 °C. GC–MS analysis identified 21 constituents, accounting for 99.99% of the total detected compounds (Table 3; Figures S3A and 4). The identified compounds (**1–21**) belonged to various chemical classes, including monoterpenes, aromatic hydrocarbons, and sesquiterpenes (Table 3). Monoterpenes dominated the oil, representing 91.75% of the total constituents, followed by aromatic hydrocarbons at 7.35% (Table 3). Eighteen monoterpene compounds (91.75%) were detected, ranging from cyclic hydrocarbons—such as α-phellandrene (**7**), α-terpinene (**8**), D-limonene (**10**), and γ-terpinene (**12**), which together formed the major fraction of the oil (62.81%)—to oxygenated cyclic compounds, including terpinen-4-ol (**17**), (–) -(1 S,2R,4R)-β-fenchol (**18**), decanal (**20**), and L-perillaldehyde (**22**) (2.24%). Oxygenated acyclic hydrocarbons, such as acetonyldimethylcarbinol (**1**), linalool (**15**), (R)-(+)-citronellal (**16**), and lauraldehyde (**26**), contributed 0.98%, while acyclic hydrocarbons, mainly α-myrcene (**6**), accounted for 8.15%. Bicyclic hydrocarbons—including 2-thujene (**2**), α-pinene (**3**), (+)-sabinene (**4**), and (+)-β-pinene (**5**)—represented 17.57% of the oil (Table 3). Aromatic hydrocarbons made up 7.35% of the total constituents, represented by o-cymene (**9**) and anthranilic acid, N-methyl-, methyl ester (**25**) (Table 3). Additionally, two sesquiterpene compounds (0.89%) were identified: a bicyclic hydrocarbon (caryophyllene, **28**) and a cyclic hydrocarbon (α-farnesene, **32**) (Table 3; Figures S3A and 4).

Egyptian *C. reticulata* Blanco variety: “Ponkan Chinese Honey Mandarin” peels gave 1.60% v/w volatile oil fresh weight, being colourless with a characteristic odor, lighter than water, clear, transparent, and not viscous at room temperature as well as at 4 °C. GC-MS analysis was used to identify a total of 19 compounds, accounting for 100.00% of all compounds found (Table 3, Figures S3B, 4). The identified compounds **1–19** belonged to different chemical classes, including monoterpene, aromatic hydrocarbon, and sesquiterpene (Table 3). Where monoterpenes represented 97.17% of the total identified compounds, followed by aromatic hydrocarbons (2.25%) (Table 3). Fourteen monoterpenes compounds (97.17%) were identified; ranging from cyclic hydrocarbon (α-terpinene **8**, D-limonene **10**, γ-terpinene **12**, p-mentha-1,4(8)-diene **13**, 69.47%) which represented the major oil fraction, to oxygenated cyclic hydrocarbon (terpinen-4-ol **17**, (-)-(1 S,2R,4R)-β-fenchol **18**, decanal 20, 2.28%), and oxygenated acyclic hydrocarbon (Linalool **15**, (R)-(+)-citronellal **16**, 2.1%), acyclic hydrocarbon (*α*-myrcene **6**, 10.21%), to bicyclic hydrocarbon (2-thujene **2**, α-pinene **3**, (+)-sabinene **4**, (+)-β-pinene **5**, 13.11%) (Table 3). While aromatic hydrocarbons represent 2.25% of the total identified compounds, in the form of *o*-cymene **9**, and anisole, 2-isopropyl-5-methyl **21** (Table 3). Also, three sesquiterpene compounds (0.58%) were identified, varying from cyclic hydrocarbon (germacrene D **29**, germacrene B **35**, 0.37%) to a cyclic hydrocarbon (tetradecanal **27**, 0.21%) (Table 3, Figures S3B, 4).

Egyptian *C. reticulata* Blanco variety: “Murcott Mandarin” peels gave 1.50% v/w volatile oil fresh weight, being colourless with a characteristic odor, lighter than water, clear, transparent, and not viscous at room temperature as well as at 4 °C. GC-MS analysis was used to identify a total of 6 compounds, accounting for 100.00% of all compounds found (Table 3, Figures S3C, 4). The identified compounds **1–6** belonged to monoterpene, class only (Table 3). Where monoterpenes represented 100.00% of the total identified compounds (Table 3). Six monoterpenes’ compounds (100.00%) were identified; ranging from cyclic hydrocarbon (D-limonene **10**, 81.13%) which represented the major oil fraction, to oxygenated cyclic hydrocarbon (decanal 20, 0.58%), and oxygenated acyclic hydrocarbon (Linalool **15**, 0.49%), acyclic hydrocarbon (*α*-myrcene **6**, 12.31%), to bicyclic hydrocarbon (α-pinene **3**, (+)-sabinene **4**, 5.49%) (Table 3).

Egyptian *C. reticulata* Blanco variety: “Clementine Mandarin” peels gave 1.50% v/w volatile oil fresh weight, being colourless with a characteristic odor, lighter than water, clear, transparent, and not viscous at room temperature as well as at 4 °C. GC-MS analysis was used to identify a total of 20 compounds, accounting for 100.00% of all compounds found (Table 3, Figures S3D, 4). The identified compounds **1–20** belonged to different chemical classes, including monoterpene, aromatic hydrocarbon, and sesquiterpene (Table 3). Where monoterpenes represented 96.85% of the total identified compounds, followed by sesquiterpene hydrocarbons (1.61%) (Table 3). Fifteen monoterpenes compounds (96.85%) were identified; ranging from cyclic hydrocarbon (α-phellandrene **7**, D-limonene **10**, γ-terpinene **12**, p-mentha-1,4(8)-diene **13**, 72.17%) which represented the major oil fraction, to oxygenated cyclic hydrocarbon (α-terpineol **19**, decanal **20**, 0.93%), and oxygenated acyclic hydrocarbon (acetonyldimethylcarbinol **1**, nonanal **14**, Linalool **15**, 1.74%), acyclic hydrocarbon (*α*-myrcene **6**, 10.73%), to bicyclic hydrocarbon (2-thujene **2**, α-pinene **3**, (+)-sabinene **4**, (+)-β-pinene **5**, 3-carene **11**, 10.97%) (Table 3). While aromatic hydrocarbon represented 1.54% of the total identified compounds, in the form of *o*-cymene **9** (Table 3). Also, four sesquiterpene compounds (1.61%) were identified, varying from cyclic hydrocarbon (germacrene D **29**) to a bi-, and tricyclic hydrocarbon (α-copaene **24**, α-cadinene **34**) and a cyclic hydrocarbon (α-farnesene **32**) (Table 3, Figures S3D, 4).

Egyptian *C. reticulata* Blanco variety: “Dancy Tangerine” peels gave 1.50% v/w volatile oil fresh weight, being colourless with a characteristic odor, lighter than water, clear, transparent, and not viscous at room temperature as well as at 4 °C. GC-MS analysis was used to identify a total of 24 compounds, accounting for 100.00% of all compounds found (Table 3, Figures S3E, 4). The identified compounds **1–24** belonged to different chemical classes, including monoterpene, aromatic hydrocarbon, and sesquiterpene (Table 3). Where monoterpenes represented 96.43% of the total identified compounds, followed by sesquiterpene hydrocarbons (2.1%) (Table 3). Eighteen monoterpenes compounds (96.43%) were identified; ranging from cyclic hydrocarbon (α-terpinene **8**, D-limonene **10**, γ-terpinene **12**, p-mentha-1,4(8)-diene **13**, 57.46%) which represented the major oil fraction, to oxygenated cyclic hydrocarbon (terpinen-4-ol **17**, (-)-(1 S,2R,4R)-β-fenchol **18**, decanal **20**, L-perillaldehyde **22**, 3.11%), and oxygenated acyclic hydrocarbon (acetonyldimethylcarbinol **1**, Linalool **15**, (R)-(+)-citronellal **16**, lauraldehyde **26**, 6.32%), acyclic hydrocarbon (*α*-myrcene **6**, 14.63%), to bicyclic hydrocarbon (2-thujene **2**, α-pinene **3**, (+)-sabinene **4**, (+)-β-pinene **5**, 3-carene **11**, 14.91%) (Table 3). While aromatic hydrocarbon represented 1.47% of the total identified compounds, in the form of Anisole, 2-isopropyl-5-methyl **21**, and thymol **23** (Table 3). Also, four sesquiterpene compounds (2.1%) were identified, varying from cyclic hydrocarbon (germacrene D **29**, germacrene A **30**, p-menth-3-ene, 2-isopropenyl-1-vinyl-, (1 S,2R)-(-)- **33**) to a tricyclic hydrocarbon (aromandendrene **31**) (Table 3, Figures S3E, 4).

In this study, mandarins, and tangerine Sp. fully mature peel essential oils are characterized by a high content of monoterpenes, 91–100% (Table 3). Where, Murcott mandarin essential oil contended only monoterpenes compounds (100.00% of the total oil) in comparing with Yousfy Balady, Ponkan Chinese honey mandarin, Clementine mandarin, and Dancy tangerine oils (91.75, 97.17, 96.85, 96.43%, respectively). The analyzed *Citrus* essential oils showed marked differences, especially from the quantitative point of view. In particular, the limonene content followed the order Murcott mandarin > Clementine mandarin > Ponkan Chinese honey mandarin > Yousfy Balady > Dancy tangerine. A marked difference was observed in 2-thujene, α-pinene, (+)-sabinene, (+)-β-pinene, α-myrcene content (Table 3). Also Dancy tangerine essential oil is characterized by a high content of oxygenated monoterpenes (9.43% of the total oil) than Ponkan Chinese honey mandarin, Yousfy Balady, Clementine mandarin, and Murcott mandarin oils (4.38, 3.22, 2.98, and 1.07%, respectively).

While Yousfy Balady essential oil is characterized by a high content of aromatic hydrocarbon (7.35% of the total oil) than Ponkan Chinese honey mandarin, Clementine mandarin, and Dancy tangerine oils (2.25, 1.54, and 1.47%, respectively) (Table 3). On the other hand, mandarins, and tangerine Sp. essential oils are characterized by a low content of sesquiterpenes < 2.10% (Table 3).

In general, Pinocarvone (22.7%), trans-pinocarveol acetate (20.0%), and β-thujone (12.8%) were the main compounds found in the Ijanikin sample, whereas citronellal (38.1%), (Z)-β-ocimene (25.9%), linalool (14.5%), and limonene (12.2%) were the main constituents found in the Ikotun sample^28^. Thirty-seven distinct components, or around 99% of the oil, were identified as essential oil ingredients in *C. reticulata* cultivated in North-East India. Limonene (46.7%), geranial (19.0%), neral (14.5%), geranyl acetate (3.9%), geraniol (3.5%), b-caryophyllene (2.6%), nerol (2.3%), neryl acetate (1.1%), etc. were the main constituents^29^. Fifty-eight essential oil compounds (97.2%) were found in *C. reticulata* cultivated in Burundi. The most prevalent chemical group (94.7%) was made up of monoterpene hydrocarbons. The most common component was limonene (84.8%), which was followed by γ-terpinene (5.4%), myrcene (2.2%), and α-pinene (1.1%). The primary components were valencene and germacrene D, with sesquiterpene hydrocarbons making up a small amount (0.2%). 2.3% were oxygenated molecules with different chemical groups. The two main chemical groupings were terpene alcohols (0.7%) and aliphatic aldehydes (0.7%). Linalool (0.7%), octanal (0.5%), and decanal (0.2%) were the principal components. The concentrations of octyl acetate, α-sinensal, decanol, and perillaldehyde were 0.1%. At less than 0.05%, thymol, α-sinensal, methyl thymol, and the acetate esters bornyl, α-terpinyl, geranyl, citronellyl, and decyl acetates were found^30^. Monoterpene level is relatively high in *C. reticulata* peel oils from Brazil, but sesquiterpene content is lower. The main constituents of *C. aurantifolia* essential oil were limonene (85.7%), ß-terpinene (6.7%), and myrcene (2.1%)^31^.

The essential oil for the tangerine species was extracted from the peels of three Spanish cultivars of C. × clementina: Fino, Loretina, and Marisol. The most prevalent monoterpene was limonene, which was followed by linalool and myrcene^32^. This outcome is consistent with Nguyen et al. (2016), who found that limonene, myrcene, and α-pinene were the primary components of essential oil extracted from Vietnam clementine peels^33^. According to a recent study by Boudries et al. (2017)^34^, the primary components of essential oils in Algerian clementine peels are limonene, β-myrcene, and sabinene. The chemical makeup of several C. × clementina cultivars from Corsica, including MA3, Nules, MA2, Hernandina, Tardia Villareal, Reina, Caffin, MacBean, Oroval, Monreal, Bruno, Tomatera, Commune, Marisol, Ragheb, and Guillermina, was previously assessed by Lota et al. 2001^35^. It was noted that the content order was limonene > myrcene > linalool. Sixty-nine components, or 99.8% of the total volatiles produced by cold pressing (CP) and supercritical CO_2_ extraction (SFE) methods, were found in the essential oil contents described in Clementine mandarin farmed in Turkey. Compared to the SFE extraction (4.2%), the CP extraction produced less oxygenated compounds (3.7%); the most common ones were carbonyls (2.09–2.10%), alcohols (1.32–1.60%), and esters (0.12–0.40%). The primary constituent is limonene (88.12–89.28%), with myrcene (4.64–3.77%) coming in second. Higher concentrations of oxygenated molecules include decanal (0.71–0.72%) and linalool (1.02–1.24%)^36^.

### GC-MS profiling of *C. aurantifolia*, and *C. limettioides* peel oils

Egyptian *C. aurantifolia* peels gave 0.5% v/w volatile oil fresh weight, being colourless with a characteristic odor, lighter than water, clear, transparent, and not viscous at room temperature as well as at 4 °C. GC-MS analysis was used to identify a total of 26 compounds, accounting for 99.06% of all compounds found (Table 4, Figures S5A, 6). The identified compounds **1–26** belonged to different chemical classes, including monoterpene, aromatic hydrocarbon, and sesquiterpene (Table 4). Where monoterpenes represented 80.45% of the total identified compounds, followed by sesquiterpene (15.88%) (Table 4). Seventeen monoterpenes compounds (80.45%) were identified; ranging from cyclic hydrocarbon (α-terpinene **7**, D-limonene **9**, γ-terpinene **11**, *p*-mentha-1,4(8)-diene **12**, 37.43%) which represented the major oil fraction, to oxygenated cyclic hydrocarbon (terpinen-4-ol **15**, α-terpineol **17**, 2.91%), and oxygenated acyclic hydrocarbon (3,7-dimethylocta-1,6-dien-3-ol **13**, α-citral **19**, nerol acetate **21**, geranyl acetate **22**, 6.94%), acyclic hydrocarbon (*α*-myrcene **6**, *α*-ocimene **10**, 3.72%), to bicyclic hydrocarbon (2-thujene **1**, α-pinene **2**, camphene **3**, (+)-sabinene **4**, (+)-β-pinene **5**, 29.45%) (Table 4). While aromatic hydrocarbon represented 2.73% of the total identified compounds, in the form of *o*-cymene **8** (Table 4). Also, eight sesquiterpene compounds (15.88%) were identified, varying from cyclic hydrocarbon (α-humulene **25**, germacrene D **26**, germacrene A **27**, germacrene B **31**, 3.58%) to a bicyclic hydrocarbon (caryophyllene **23**, cis-α-bergamotene **24**, aromandendrene **29**, 5.87%), and a cyclic hydrocarbon (α-farnesene **30**, 6.43%) (Table 4, Figures S5A, 6).

Egyptian *C. limettioides* peels gave 0.7% v/w volatile oil fresh weight, being colourless with a characteristic odor, lighter than water, clear, transparent, and not viscous at room temperature as well as at 4 °C. GC-MS analysis was used to identify a total of **15** compounds, accounting for 99.27% of all compounds found (Table 4, Figures S5B, 6). Using GC-MS analysis, *C. limettioides* identified compounds **1–15** belonged also to monoterpene, and sesquiterpene (Table 4). Where monoterpenes represented 92.06% of the total identified compounds, followed by sesquiterpene (7.21%) (Table 4). Eleven monoterpenes compounds (92.06%) were identified; ranging from cyclic hydrocarbon (D-limonene **9**, 64.15%) which represented the major oil fraction, to oxygenated acyclic hydrocarbon (3,7-dimethylocta-1,6-dien-3-ol **13**, (R)-(+)-citronellal **14**, (R)-(+)-citronellol **18**, citronellol acetate **20**, 10.27%), oxygenated bicyclic hydrocarbon ((-)-(1 S,2R,4R)-β-fenchol **16**, 0.49**%**), acyclic hydrocarbon (*α*-myrcene **6**, *α*-ocimene **10**, 11.93%), to bicyclic hydrocarbon (2-thujene **1**, α-pinene **2**, (+)-sabinene **4**, 5.22%) (Table 4). Also, four sesquiterpene compounds (7.21%) were identified, varying from cyclic hydrocarbon (germacrene D **26**, cis-α-bisabolene **28**, 4.88%) to a bicyclic hydrocarbon (caryophyllene **23**, cis-α-bergamotene **24**, 2.33%) (Table 4, Figures S5B, 6).

Particularly in green fruits, a decrease in oxygenated monoterpenes, and an increase in monoterpene hydrocarbons were observed during ripening^37–39^. In this study, *C. limettioides* essential oil is characterized by a high content of oxygenated monoterpenes (10.76% of the total oil) than *C. aurantifolia* oils (9.85%). The analyzed *Citrus* essential oils showed marked differences especially from the quantitative point of view, depending on the ripening stage of fruits. In particular, the limonene content followed the order *C. limettioides* > *C. aurantifolia*. A marked difference was observed in 2-thujene, α-pinene, (+)-sabinene, (+)-β-pinene, α-myrcene content, (Table 4).

On the other hand, a marked difference was observed in Sesquiterpene hydrocarbon essential oils content in the following order *C. aurantifolia* > *C. limettioides* (15.88, 7.21%, respectively) (Table 4).

According to the literature, the Brazil *C. aurantifolia* peels volatile oil differently from the Egyptian, having 17 compounds, where monoterpene (94.00%), represents mainly limonene (77.50%), myrecene (4.40%), citronellal (3.20%), and linalool (3.50%), while sesquiterpene (4.90%) represents mainly germacrene D (1.50%), and *β-*bisabolene (1.50%)^40^. The essential oil constituents reported in *C. aurantifolia* grown in Mexicana contained 55 compounds, representing 99.60% of the whole oil. Of these, limonene (53.8%), ɣ-terpinene (16.50%), and β-pinene (12.60%) were the most abundant^41^. The essential oil constituents reported in *C. aurantifolia* grown in Italy contained 55 compounds, limonene was the main constituent in (53.76%) peel oil. Aliphatic aldehydes ketones, esters, and alcohols were also detected in the oil^19^. *C. aurantifolia* peel oils (Cosenza (Italy)) are characterized by a very high content of monoterpenes, whereas sesquiterpenes were less represented. *C. aurantifolia* essential oil was characterized by 49 constituents (93.6% of the total oil) in which the dominant components were the monoterpene hydrocarbon limonene (49.2%), β-pinene (14.1%), γ -terpinene (6.6%), and β-myrcene (3.1%). Among identified sesquiterpene, β-bisabolene (2.4%) represents the most abundant constituent^42^. The Ben Tre province *C. aurantifolia* peel oil showed quite similarity in the content to the Egyptian one. The principally identified components were limonene (62.17%), β-pinene (11.72%), γ-terpinene (12.356%)^43^. The extraction method, too, influences the concentration and composition of essential oils. The essential oil constituents of Mexicana *C. aurantifolia* were reported to contain 35 constituents using cold-pressed. The major components of the key lime oil were limonene, γ -terpinene, β-pinene, geranial, 5,7-dimethoxycoumarin, and bergaptene^44^.

The Mexico *C. limettioides* peels volatile oil differently from the Egyptian, having 25 compounds, where citronellal (3.80%) was found as major compound^45^. The essential oil constituents reported in *C. limettioides* grown in USA contained 24 compounds, the main constituents were DL-limonene, myrcene, (+)-sabinene, and triacontane, respectively^46^. Also, the essential oil constituents reported in *C. limettioides* grown in Hisar contained fourteen different components constituting approximately ≥ 99% of the oil. The major components were dl-limonene (89.089%), β-myrcene (2.933%), (±)-linalool (2.927%), α-pinene (0.865%), (E)-citral (0.749%) etc^47^., .

### GC-MS profiling of *Citrus paradisi* Macf. peel oil

Egyptian *Citrus paradisi* Macf. peels gave 1.1% v/w volatile oil fresh weight, being colourless with a characteristic odor, lighter than water, clear, transparent, and not viscous at room temperature as well as at 4 °C. GC-MS analysis was used to identify a total of 22 compounds, accounting for 99.74% of all compounds found (Table 5, Figures S7-8). The identified compounds **1–22** belonged to different chemical classes, including monoterpene, and sesquiterpene (Table 5). Where monoterpenes represented 91.84% of the total identified compounds, followed by sesquiterpene (7.9%) (Table 5). Fourteen monoterpenes compounds (91.84%) were identified; ranging from cyclic hydrocarbon (D-limonene **4**, γ-terpinene **6**, 63.61%) which represented the major oil fraction, to oxygenated cyclic hydrocarbon (*cis*-linalool oxide **7**, α-terpineol **11**, 1.38%), and oxygenated acyclic hydrocarbon (pelargonaldehyde **8**, 3,7-dimethylocta-1,6-dien-3-ol **9**, (R)-(+)-citronellal **10**, capraldehyde **12**, α-citral **13**, geranyl acetate **14**, 4.92%), acyclic hydrocarbon (*α*-myrcene **3**, *α*-ocimene **5**, 14.7%), to bicyclic hydrocarbon (*α*-pinene **1**, sabinene **2**, 7.23%) (Table 5).

Also, eight sesquiterpene compounds (7.9%) were identified, varying from cyclic hydrocarbon (germacrene D **16**, α-humulene **18**, germacrene B **19**, 2.33%) to a bicyclic hydrocarbon (caryophyllene **17**, α-cadinene **20**, 3.82%), oxygenated bicyclic hydrocarbon ((1 S,4 S,7R,8 S)-1,4a-dimethyl-7-(prop-1-en-2-yl) decahydronaphthalen-1-ol **21**, nootkatone **22**, 0.74%), and a tricyclic hydrocarbon (*α*-copaene **15**, 1.01%) (Table 5).

The Sudan *C. paradisi* peels volatile oil differently from the Egyptian, having 12 compounds, where monoterpene (95.51%), represents mainly limonene (74.45%), myrecene (12.85%), and *α*-pinene (3.74%), while sesquiterpene (4.08%) represents mainly *trans-*caryophyllene (1.15%), and *α*-farnesene (1.13%)^48^. The essential oil constituents reported in *C. paradisi* grown in Barcelona contained 25 compounds, representing 96% of the whole oil. Of these, limonene (96.2%) and myrcene (1.5%) were the most abundant. At trace levels (0.06%), we found α-thujene, *β*-pinene, *β*-ocymene, nonanal, isogeraniol, trans-limonene oxide, α-terpineol, trans-carveol, carvone, citral, geranil acetate, germacrene D and linalool^49^. The essential oil constituents reported in *C. paradisi* grown in Nigeria contained 15 compounds, limonene was the main constituent in (94.2%) peel oil. Aliphatic aldehydes and alcohols were also detected in the oil^50^. The México *C. paradisi* peel oil showed quite similarity to the Egyptian one. The principally identified components were limonene (94.427%), myrcene (1.852%), α-pinene (0.544%), sabinene (0.340%), decanal (0.211%), citral (0.104%), linalool (0.085%), *β*-pinene (0.065%), γ-terpinene (0.049%), 1-terponen-4-ol (0.012%) nootkatone (0.009%)^51^.

The extraction method, too, influences the concentration and composition of essential oils. The essential oil constituents of Turkish *C. paradisi* were reported to contain 27 constituents using cold-pressed. The major components of the grapefruit oil were limonene, myrcene, β-pinene, sabinene and β-caryophyllene, which constituted altogether about 96.5% of the whole oil^52^. Also, Algeria *C. paradisi* was reported to contain 19, 18, and 20 constituents using microwave hydro-diffusion and gravity, hydro-distillation, and cold pressing, respectively. Where limonene, and myrcene represented the major oil fraction range 91.50–93.00% of the whole oil^53^.

### Multivariate and statistical analysis

#### Unsupervised multivariate analyses

##### Principal component analysis (PCA)

**Fig. 1:**
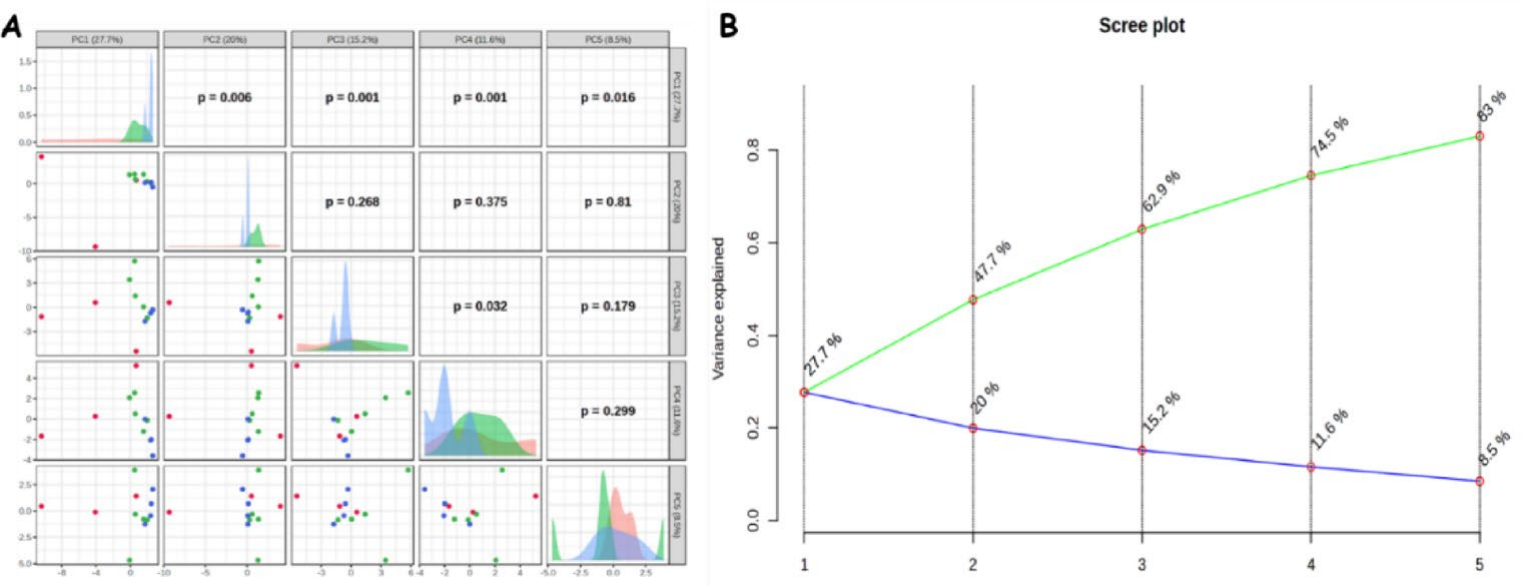
(**A**) PCA pairwise score plot of the unsupervised method; (**B**) PCA scree plot of the unsupervised method.

To get an overview of the metabolic differences between the groups under analysis, PCA was first used. Five PCA components (PCs) accounted for 83% of the total variation, as seen in the PCA pairwise score and scree plots (Fig. 1). The first and second PCs contributed 47.7% of the total variation (PC1 and PC2 represent 27.7 and 20%, respectively), while the first, second, and three PCs represented 62.9% of the total variation (PC1, PC2, and PC3 represent 27.7, 20, and 15.2%, respectively). (Fig. 1B).

**Fig. 2:**
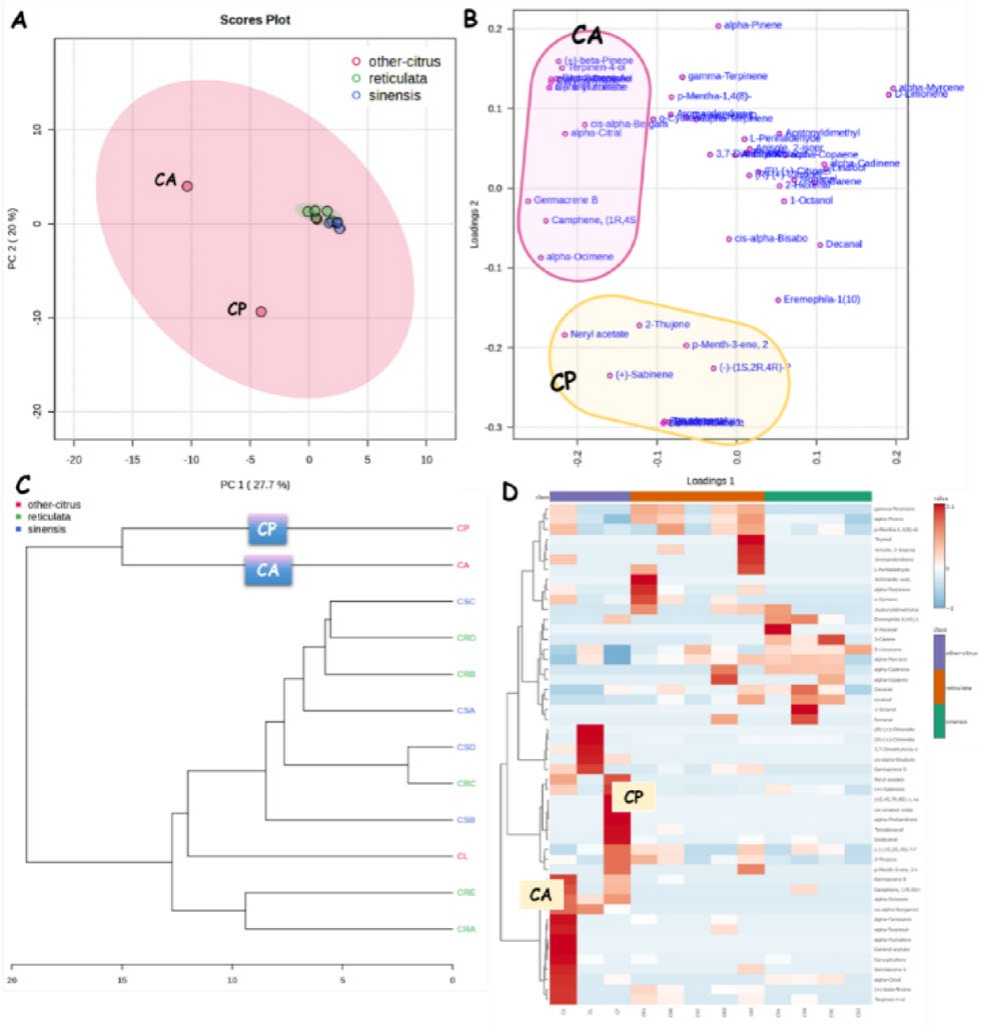
Unsupervised Multivariate Analyses. (**A**) 2D PCA scores plot of the unsupervised method; (**B**) 2D PCA loadings plot of the unsupervised method; (**C**) HCA plot showed as dendogram; (**D**) heatmap showing the metabolites pattern responsible for the variation of the samples CA and CP. (CA) *Citrus aurantifolia* Swingle: “Key Lime”; (CP) *Citrus paradisi* Macf.: “Tangelo Seminole”; (other *Citrus*) *C. aurantifolia*, *C. limettioides*, and *C. paradisi*; reticulata *(C. reticulata* varieties); and sinensis *(C. sinensis* varieties).

The three *Citrus* groups (*C. sinensis*, *C. reticulata*, and other *Citrus* (*C. aurantifolia*, *C. limettioides*, and *C. paradisi*) were well separated in the score plot (Fig. 2A), showing different metabolic profiles. The analyzed groups were primarily divided into three distinct sections between PC1 and PC2 in the PCA 2D scores plot (Fig. 2A); *Citrus aurantifolia* Swingle: “Key Lime” (CA) oil and *Citrus paradisi* Macf.: “Tangelo Seminole” (CP) oil were the outliers. All *Citrus* groups were found to be on the PC1 positive side, with the exception of *Citrus aurantifolia* Swingle: “Key Lime” (CA) oil, which was on the negative side. Regarding the main component PC2, *Citrus aurantifolia* Swingle: “Key Lime” (CA) oil is locaed on the positive side while *Citrus paradisi* Macf.: “Tangelo Seminole” (CP) oil is on the negative side (Fig. 2A). Consequently, the PCA scores plot revealed the dispersion of *C*. *aurantifolia* swingle (key lime) (CA) and *C*. *paradisi* Macf. (Tangelo seminole) (CP) oils, which is further supported from their distinct patterns in the heatmap plot (Fig. 2D). The PCA loadings plot (Fig. 2B) highlighted high level of the monoterpenes, 2-thujene, (+)-sabinene, α-phellandrene, dodecanal, tetradecanal, germacrene B, neryl acetate, cis-linalool oxide, and (1 S,4 S,7R,8 S)-1,4a-dimethyl-7-(prop-1-en-2-yl) decahydronaphthalen-1-ol, as the identified metabolites contributed to the variation of the anomalous *C. paradisi* oil. Also, highlighted terpinen-4-ol, α-citral, caryophyllene, (+)-β-pinene, and α-farnesene, as the major metabolites contributed to the variation of *C. aurantifolia* oil.

##### Hierarchical clustering analysis (HCA)

PCA results were corroborated by hierarchical clustering analysis (HCA) (Fig. 2C). *Citrus* samples were divided into two major clusters, according to the dendrogram. The consistency of the PCA separation was confirmed by the first cluster, which displayed *C. aurantifolia* (CA) and *C. paradisi* (CP) separated from other *Citrus* samples. However, the heatmap (Fig. 2D), which showed clear concentration differences in discriminant metabolites between groups, provided a thorough summary of metabolite distributions and abundance. Red signals indicated higher abundance, while blue signals indicated lower levels.

##### Self-organizing map (SOM) analysis

**Fig. 3:**
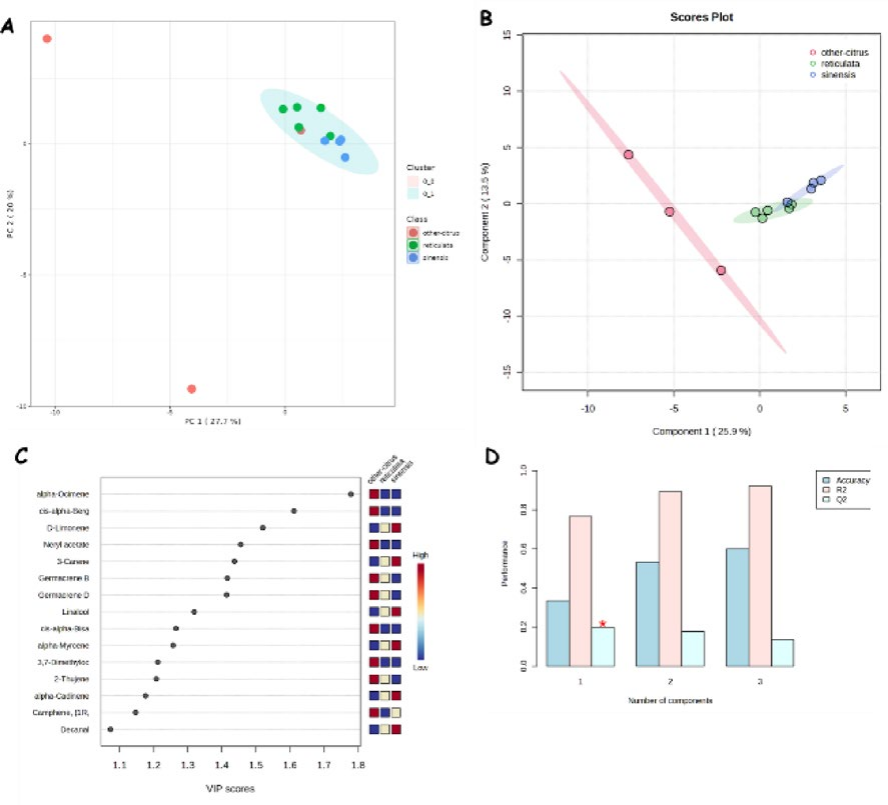
Unsupervised and supervised Multivariate Analyses. (**A**) Self-Organizing Map (SOM) analysis of the unsupervised method; (**B**) PLS-DA scores plot of the supervised method; (**C**) VIP score plot of PLS-DA; (**D**) PLS-DA cross validation models. ((other *Citrus*) *C. aurantifolia*, *C. limettioides*, and *C. paradisi*; reticulata *(C. reticulata* varieties); and sinensis *(C. sinensis* varieties).

Another unsupervised technique for visualising complex nonlinear interactions was SOM analysis. The PCA and HCA results were supported by the SOM map, which showed discrete clusters that matched the sample groupings (Fig. 3A).

#### Supervised multivariate analyses

To improve group separation and find discriminatory metabolites, supervised multivariate models (PLS-DA and sPLS-DA) were used. The distinction seen in PCA was further validated using PLS-DA and sPLS-DA.

##### Partial least squares–discriminant analysis (PLS-DA)

Compared to PCA, PLS-DA score plots demonstrated superior separation. Other *Citrus* species (C. aurantifolia, C. limettioides, and C. paradisi) were clearly distinguished from C. reticulata and C. sinensis variations in the PLS-DA scores plot (Fig. 3B), whereas the latter two groups remained closely clustered, suggesting metabolic similarities. The model’s reliability was validated by model validation statistics (R² Q²) (Fig. 3D); R² values showed acceptable accuracy across the number of components showing adequate performance. Although the model well explains variation between groups, Q² values indicated a modest predictive capacity, indicating that external validation with a larger sample size may be required to establish its robustness.

On the other hand, Variable Importance in Projection (VIP) analysis (Fig. 3C) identified the key discriminant metabolites causing this separation. The compounds with the highest VIP scores were α-ocimene, cis-α-bergamotene, D-limonene, neryl acetate, 3-carene, and germacrene B. These metabolites represent the most influential markers contributing to varietal differentiation, with D-limonene being especially relevant due to their well-known roles in *Citrus* aroma profiles.

#### Sparse PLS-DA (sPLS-DA)

**Fig. 4:**
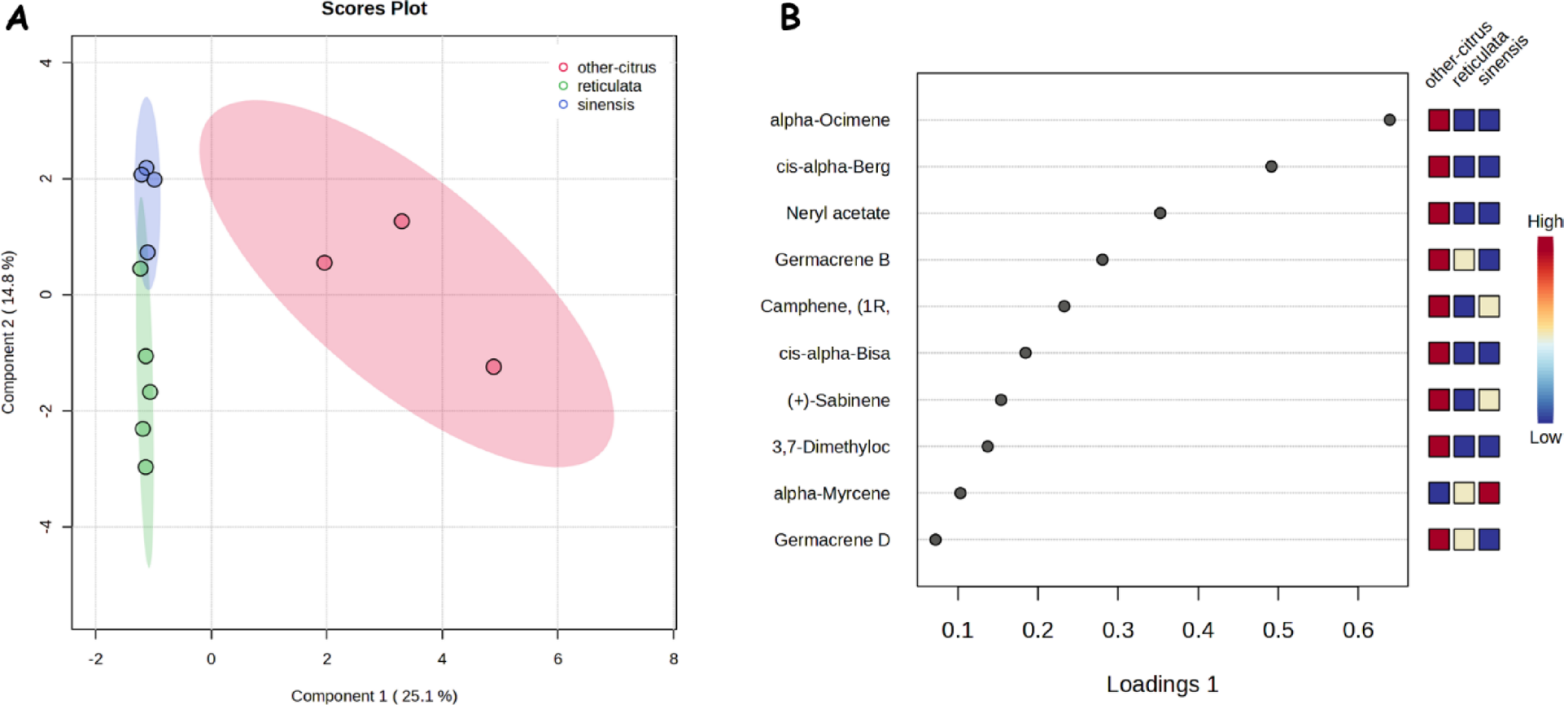
Supervised Multivariate Analyses. (**A**) sPLS-DA scores plot of the supervised method (**B**) sPLS-DA loadings plot of the supervised method. Red color: other *Citrus* (*C. aurantifolia*, *C. limettioides*, and *C. paradisi*); Green color: reticulata *(C. reticulata* varieties); and Blue color: sinensis *(C. sinensis* varieties).

sPLS-DA was used to reduce dimensionality and choose the most discriminative metabolites. By optimizing variable selection, the sPLS-DA scores plot (Fig. 4A) improved separation; distinct group discrimination was seen; other *Citrus* varieties (*C. aurantifolia*, *C. limettioides*, and *C. paradisi*) could be distinguished from each other as well as from *C. reticulata* and *C. sinensis*. A subset of metabolites that significantly contributed to group differentiation was found by the sPLS-DA loading plot, offering potential biomarker candidates (Fig. 4B). The effectiveness of sparse modelling in concentrating on a smaller collection of discriminant metabolites while reducing noise is shown in the greater discrimination attained in the sPLS-DA model.

## Discussion

In this study, the volatile profiles of *C. sinensis* varieties combine with the volatile profiles of *C. reticulata* Blanco, *C. aurantifolia*,* C. limettioides*, and *C. paradisi*, in the presence of sporadically terpenoid compounds with comparable proportions, such as α-pinene **4**, (+)-sabinene **5**, α-myrcene **6**, D-limonene **8**, γ-terpinene **11**, (R)-(+)-citronellal **16**, and caryophyllene **23**, consistent with what was previously reported by González-Mas et al., 2019^10^. Beside, the EOs identified from *C. sinensis* varieties seem to differ principally from the others by presence of some exclusive terpenoid compounds, such 2-hexenal **2** (washington navel (Navelina)), 1-octanol **14** (balady seeded orange), Eremophila-1(10),11-diene **24** (washington navel (Navelina), balady seeded orange, and blood orange) (Table 2, Figures S1-2). Moreover, several other nonterpenoid aldehyde compounds have been mainly founded in the peel of *C. sinensis* varieties and only sporadically in the peel of other *Citrus* species. This is the case of nonanal **13**, decanal **19**, and dodecanal **22**, comparing with *C. reticulata* Blanco, *C. aurantifolia*,* C. limettioides*, and *C. paradisi* varieties (Tables 2, 3, 4 and 5). Among other compounds described in rinds of *C. sinensis*, the monoterpenes 2-thujene **3**, and *p*-mentha-1,4(8)-diene **12**, (-)-(1 S,2R,4R)-*β*-fenchol **18**, which have only been reported in the species of *C. reticulata*, *C. aurantifolia*, and *C. limettioides* according to their volatile profile (Tables 2, 3, 4 and 5). And the sesquiterpene hydrocarbons α-copaene **21**, and α-cadinene **25** which have only been reported in the species of *C. reticulata*, and *C. paradisi* according to their volatile profile (Tables 2, 3, 4 and 5), consistent with what was previously reported by González-Mas et al., 2019^10^. According to literature, limonene is usually reported between 90 and 97% in *C. sinensis*, although this percentage decreased down to 64% in some studies^10^. But this differ from what was stated in this study, where limonene was down to 61.95% (balady seeded orange) (Table 2).

**Table 2:** *Citrus sinensis* varieties: “Washington Navel (Navelina)”, “Balady Seeded Orange”, “Blood Orange”, and “Sweet Orange Sukkari”, peel oils composition using GC/MS analysis.

Nu.	Identified Compound	MF	%Area				RT	RI
			Washington navel (Navelina)	Balady seeded orange	Blood orange	Sweet orange Sukkari		
1	Acetonyldimethylcarbinol	C_6_H_12_O_2_	0.21				2.55	912
2	2-Hexenal	C_6_H_10_O	0.17				2.68	930
3	2-Thujene	C_10_H_16_	0.45		0.53		4.10	935
4	*α*-Pinene	C_10_H_16_	5.02	5.03	4.89	3.14	4.23	924
5	(+)-Sabinene	C_10_H_16_	4.21	1.99	2.42		4.96	953
6	*α*-Myrcene	C_10_H_16_	13.72	14.82	14.29	4.59	5.35	981
7	α-Phellandrene	C_10_H_16_		0.38			5.64	929
8	D-Limonene	C_10_H_16_	67.55	61.95	63.51	91.23	6.25	933
9	3-Carene	C_10_H_16_	2.00	1.11	3.76		6.74	898
10	Camphene, (1R,4 S)-(+)-	C_10_H_16_		0.13			6.76	900
11	*γ-*Terpinene	C_10_H_16_	0.21				7.00	931
12	*p-*Mentha-1,4(8)-diene	C_10_H_16_	0.46	0.26	0.76		7.76	923
13	Nonanal	C_9_H_18_O		0.20			7.93	903
14	1-Octanol	C_8_H_18_O		0.30			7.22	903
15	Linalool	C_10_H_18_O	1.09	6.29	5.52	1.04	8.02	923
16	(R)-(+)-Citronellal	C_10_H_18_O	0.33	0.42	0.36		9.34	948
17	Terpinen-4-ol	C_10_H_18_O		0.27			10.16	933
18	(-)-(1 S,2R,4R)-*β*-Fenchol	C_10_H_18_O		0.86	0.64		10.52	940
19	Decanal	C_10_H_20_O	1.06	2.30	0.95		10.99	936
20	α-Citral	C_10_H_16_O	1.17	1.06	1.43		11.87	900
21	α-Copaene	C_15_H_24_			0.18		16.63	939
22	Dodecanal	C_12_H_24_O		0.33			17.32	964
23	Caryophyllene	C_15_H_24_	0.23				17.82	939
24	Eremophila-1(10),11-diene	C_15_H_24_	1.90	2.07	0.55		19.98	947
25	α-Cadinene	C_15_H_24_	0.22	0.21	0.21		20.84	959
Monoterpenes hydrocarbon			93.62	85.67	90.16	98.96		
Oxygenated monoterpenes hydrocarbon			4.03	12.03	8.9	1.04		
Sesquiterpene hydrocarbon			2.35	2.28	0.94			
Total			**100.00**	**99.98**	**100.00**	**100.00**		

RI: retention index relative to *n*-alkanes, RT: retention time (min), MF: molecular formula, : major compound, %: percentage.

The volatile profiles of *C. reticulata* Blanco varieties combine with the volatile profiles of *C. sinensis*,* C. aurantifolia*,* C. limettioides*, and *C. paradisi*, in the presence of sporadically terpenoid compounds with comparable proportions, such as α-pinene **3**, (+)-sabinene **4**, α-myrcene **6**, D-limonene **10**, γ-terpinene **12**, (R)-(+)-citronellal **16**, and caryophyllene **28**, consistent with what was previously reported^10^. Beside, the EOs identifed from *C. reticulata* Blanco varieties seem to differ principally from the others by presence of some exclusive aromatic hydrocarbon, terpenoid, and non-terpenoid compounds, such anisole, 2-isopropyl-5-methyl **21** (ponkan chinese honey), L-perillaldehyde **22**, thymol **23** (dancy tangerine), anthranilic acid, N-methyl-, methyl ester **25** (yousfy balady), tetradecanal **27** (ponkan chinese honey), and *p*-menth-3-ene, 2-isopropenyl-1-vinyl-, (1 S,2R)-(-)- **33** (dancy tangerine) (Table 3, Figures S3-4). Moreover, several other nonterpenoid aldehyde compounds have been mainly founded in the peel of *C. reticulata* Blanco varieties and only sporadically in the peel of other *Citrus* species. This is the case of nonanal **14**, decanal **20**, and dodecanal **26**, comparing with *C. sinensis*,* C. aurantifolia*,* C. limettioides*, and *C. paradisi* varieties (Tables 2, 3, 4 and 5). Among other compounds described in rinds of *C. reticulata*, the monoterpenes 2-thujene **2**, and *p*-mentha-1,4(8)-diene **13**, (-)-(1 S,2R,4R)-*β*-fenchol **18**, which have only been reported in the species of *C. sinensis*, *C. aurantifolia*, and *C. limettioides* according to their volatile profile (Tables 2, 3, 4 and 5). And the sesquiterpene hydrocarbons α-copaene **24**, and α-cadinene **34** which have only been reported in the species of *C. sinensis*, and *C. paradisi* according to their volatile profile (Tables 2, 3, 4 and 5), consistent with what was previously reported by González-Mas et al., 2019^10^. According to literature, from a quantitative perspective, although non-terpenoid aldehyde compounds could help to differentiate this species from others, the most abundant compounds in *C. reticulata* EO are monoterpene hydrocarbons^10^. Among them, the most relevant is limonene, usually representing about 95% of the total EO, but occasionally down to 60% in some studies^54^. The next compounds in abundance are ɣ-terpinene, sometimes reaching values above 15%^54^, α-myrcene (7.43–0.1%)^54^, α-pinene (3.93–0.1%), or *β*-pinene (4%-traces)^54^. Monoterpenoids, and sesquiterpene linalool, *β*-citronellal, and α-sinensal can reach up to 2.9%, 0.6%, and 0.7 − 0.1%, respectively^55^. But this differ from what was stated in this study, where limonene was down to 32.87% (dancy tangerine), α-myrcene (8.15–14.63%), α-pinene (4.55–8.46%), or *β*-pinene (2.38–5.60%). While monoterpenoids linalool and *β*-citronellal can reach up to 5.56% and 0.38%, respectively, with complete absence of α-sinensal (Table 3).

**Table 3:** *Citrus reticulata* Blanco varieties: “Yousfy Balady”, “Ponkan Chinese Honey Mandarin”, “Murcott Mandarin”, “Clementine Mandarin”, and “Dancy Tangerine”, peel oils composition using GC/MS analysis.

Nu.	Identified Compound	MF	Area %					RT	RI
			Mandarin			Tangerine			
			*Yousfy Balady*	*Ponkan Chinese honey*	*Murcott*	*Clementine mandarin*	*Dancy tangerine*		
1	Acetonyldimethylcarbinol	C_6_H_12_O_2_	0.23			0.12	0.14	2.55	912
2	2-Thujene	C_10_H_16_	3.30	2.05		1.06	2.02	4.10	935
3	*α*-Pinene	C_10_H_16_	7.64	6.81	4.55	6.27	8.46	4.23	924
4	(+)-Sabinene	C_10_H_16_	1.03	1.10	0.94	1.01	1.26	4.96	953
5	(+)-*β*-Pinene	C_10_H_16_	5.60	3.15		2.38	2.88	5.03	929
6	*α*-Myrcene	C_10_H_16_	8.15	10.21	12.31	10.73	14.63	5.35	981
7	α-Phellandrene	C_10_H_16_	0.27			0.25		5.64	929
8	*α-*Terpinene	C_10_H_16_	3.69	0.44			1.15	5.93	939
9	*o-*Cymene	C_10_H_14_	5.68	1.87		1.54		6.01	933
10	D-Limonene	C_10_H_16_	36.62	47.42	81.13	54.69	32.87	6.25	933
11	3-Carene	C_10_H_16_				0.25	0.29	6.74	898
12	*γ-*Terpinene	C_10_H_16_	22.23	19.66		16.05	21.63	7.00	931
13	*p-*Mentha-1,4(8)-diene	C_10_H_16_		1.95		1.18	1.81	7.76	923
14	Nonanal	C_9_H_18_O				0.13		7.93	903
15	Linalool	C_10_H_18_O	0.38	1.83	0.49	1.49	5.56	8.02	923
16	(R)-(+)-Citronellal	C_10_H_18_O	0.19	0.27			0.38	9.34	948
17	Terpinen-4-ol	C_10_H_18_O	0.48	0.35			0.36	10.16	933
18	(-)-(1 S,2R,4R)-β-Fenchol	C_10_H_18_O	0.87	0.82			1.07	10.52	940
19	α-Terpineol	C_10_H_18_O				0.38		10.53	945
20	Decanal	C_10_H_20_O	0.65	1.11	0.58	0.55	1.28	10.99	936
21	Anisole, 2-isopropyl-5-methyl	C_11_H_16_O		0.38			1.14	11.89	846
22	L-Perillaldehyde	C_10_H_14_O	0.24				0.40	12.70	871
23	Thymol	C_10_H_14_O					0.33	13.89	917
24	α-Copaene	C_15_H_24_				0.31		16.63	939
25	Anthranilic acid, N-methyl-, methyl ester	C_9_H_11_NO_2_	1.67					16.79	944
26	Dodecanal	C_12_H_24_O	0.18				0.24	17.32	964
27	Tetradecanal	C_14_H_28_O		0.21				17.34	916
28	Caryophyllene	C_15_H_24_	0.26					17.82	939
29	Germacrene D	C_15_H_24_		0.19		0.45	0.61	19.58	958
30	Germacrene A	C_15_H_24_					0.23	20.27	925
31	Aromandendrene	C_15_H_24_					0.84	20.36	897
32	α-Farnesene	C_15_H_24_	0.63			0.81		20.55	903
33	p-Menth-3-ene, 2-isopropenyl-1-vinyl-, (1 S,2R)-(-)-	C_15_H_24_					0.42	20.72	904
34	α-Cadinene	C_15_H_24_				0.35		20.84	959
35	Germacrene B	C_15_H_24_		0.18				21.70	947
Monoterpenes hydrocarbon			88.53	92.79	98.93	93.87	87.00		
Oxygenated monoterpenes hydrocarbon			3.22	4.38	1.07	2.98	9.43		
Aromatic hydrocarbon			7.35	2.25		1.54	1.47		
Sesquiterpene hydrocarbon			0.89	0.37		1.61	2.1		
Oxygenated sesquiterpene hydrocarbon				0.21					
**Total**			**99.99%**	**100.00%**	**100.00%**	**100.00%**	**100.00%**		

RI: retention index relative to *n*-alkanes, RT: retention time (min), MF: molecular formula, : major compound, %: percentage.

The volatile profiles of *C. aurantifolia*, and *C. limettioides* varieties combine with the volatile profiles of *C. sinensis*,* C. reticulata* Blanco, and *C. paradisi*, in the presence of sporadically terpenoid compounds with comparable proportions, such α-pinene **2**, (+)-sabinene **4**, α-myrcene **6**, D-limonene **9**, and caryophyllene **23**, consistent with what was previously reported^10^. While, γ-terpinene **11**, (R)-(+)-citronellal **14** were present only in *C. aurantifolia*, and *C. limettioides* varieties, respectively, in combine with the other varites (Tables 2, 3, 4 and 5). Beside, the EOs identifed from *C. aurantifolia*, and *C. limettioides* varieties seem to differ principally from the others by presence of some exclusive terpenoid compounds, such (R)-(+)-citronellol **18**, citronellyl acetate **20** (*C. limettioides*), neryl acetate **21**, geranyl acetate **22** (*C. aurantifolia*), *cis-α*-bergamotene **24** (*C. aurantifolia*, and *C. limettioides*), and *cis-α*-bisabolene **28** (*C. limettioides*) (Table 4, Figures S5-6). Among other compounds described in rinds of *C. aurantifolia*, and *C. limettioides*, the monoterpenes 2-thujene **1**, which has only been reported in the species of *C. sinensis*, and *C. reticulata* according to their volatile profile (Tables 2, 3, 4 and 5). And the sesquiterpene hydrocarbon germacrene D **26** which have only been reported in the species of *C. reticulata* Blanco, and *C. paradisi* according to their volatile profile (Tables 2, 3, 4 and 5), consistent with what was previously reported by González-Mas et al., 2019^10^.

As reported in the previous studies, the percentage of limonene may drop to 39.9% in the oil of *C. aurantifolia*, with the abundance of other terpene compounds is increased, such as β-pinene, neryl acetate, geranyl acetate, β-bisabolene, (E)-α-bergamotene, germacrene D and β-caryophyllene^56^. This is consistent with what was stated in this study (Table 4), where limonene was down to 20.72%, with the abundance of other terpene compounds as α & β-pinene, neryl acetate, geranyl acetate, (E)-α-bergamotene, germacrene B & D, and β-caryophyllene, with the absence of β-bisabolene.

**Table 4:** *C. aurantifolia*, and *C. limettioides* peel oils composition using GC/MS analysis.

Nu.	Identified Compound	MF	%Area		RT	RI
			*C. aurantifolia*	*C. limettioides*		
1	2-Thujene	C_10_H_16_	1.41	0.23	4.10	935
2	*α*-Pinene	C_10_H_16_	6.49	4.17	4.23	924
3	Camphene, (1R,4 S)-(+)-	C_10_H_16_	0.37		4.47	951
4	(+)-Sabinene	C_10_H_16_	6.06	0.82	4.96	953
5	(+)-*β*-Pinene	C_10_H_16_	15.12		5.03	929
6	*α*-Myrcene	C_10_H_16_	2.60	11.51	5.35	981
7	*α-*Terpinene	C_10_H_16_	0.85		5.93	939
8	*o-*Cymene	C_10_H_14_	2.73		6.01	933
9	D-Limonene	C_10_H_16_	20.72	64.15	6.25	933
10	*α*-Ocimene	C_10_H_16_	1.12	0.42	6.72	930
11	*γ-*Terpinene	C_10_H_16_	14.33		7.00	931
12	*p-*Mentha-1,4(8)-diene	C_10_H_16_	1.53		7.76	923
13	3,7-Dimethylocta-1,6-dien-3-ol	C_10_H_18_O	0.41	1.96	8.01	925
14	(R)-(+)-Citronellal	C_10_H_18_O		6.32	9.34	948
15	Terpinen-4-ol	C_10_H_18_O	1.52		10.16	933
16	(-)-(1 S,2R,4R)-β-Fenchol	C_10_H_18_O		0.49	10.52	940
17	α-Terpineol	C_10_H_18_O	1.39		10.53	945
18	(R)-(+)-Citronellol	C_10_H_20_O		1.49	11.85	921
19	α-Citral	C_10_H_16_O	5.19		11.86	908
20	Citronellyl acetate	C_12_H_22_O_2_		0.50	15.70	913
21	Neryl acetate	C_12_H_20_O_2_	0.42		15.95	924
22	Geranyl acetate	C_12_H_20_O_2_	0.92		16.51	935
23	Caryophyllene	C_15_H_24_	3.05	0.27	17.82	939
24	*cis*-α-Bergamotene	C_15_H_24_	2.47	2.06	18.46	948
25	α-Humulene	C_15_H_24_	0.34		18.80	927
26	Germacrene D	C_15_H_24_	0.77	1.92	19.58	958
27	Germacrene A	C_15_H_24_	0.72		20.27	925
28	cis-*α*-Bisabolene	C_15_H_24_		2.96	20.36	939
29	Aromandendrene	C_15_H_24_	0.35		20.36	897
30	α-Farnesene	C_15_H_24_	6.43		20.55	903
31	Germacrene B	C_15_H_24_	1.75		21.70	947
Monoterpenes hydrocarbon			70.60	81.3		
Oxygenated monoterpenes hydrocarbon			9.85	10.76		
Aromatic hydrocarbon			2.73			
Sesquiterpene hydrocarbon			15.88	7.21		
**Total**			**99.06%**	**99.27%**		

RI: retention index relative to *n*-alkanes, RT: retention time (min), MF: molecular formula, : major compound, %: percentage.

Acoording to this study, the volatile profile from *C. paradisi* is very close to those of *C. sinensis*, *C. reticulata* Blanco, *C. aurantifolia*, and *C. limettioides* (Tables 2, 3, 4 and 5), in the presence of sporadically terpenoid compounds with comparable proportions, such α-pinene **1**, (+)-sabinene **2**, α-myrcene **3**, D-limonene **4**, γ-terpinene **6**, (R)-(+)-citronellal **10**, and caryophyllene **17**, consistent with what was previously reported^10^. According to literature, the major compounds in *C. paradisi* are often with lower levels of γ -terpinene and linalool compare to those with *C. sinensis* and *C. reticulata*^57^, and with higher levels of nootkatone, which can reach 0.2% in *C. paradisi*^58^, while in the other two varieties they usually appear in lower percentages^59^. This is consistent with what was stated in this study (Table 5), concerning γ -terpinene (Tables 2 and 3), but differ with the level of nootkatone which count for 0.51%, with absence of linalool, and presence *cis*-linalool oxide instead (Table 5).

**Table 5:** *Citrus paradisi* Macf. peel oil composition using GC/MS analysis.

Nu.	Identified Compound	MF	Area %	RT	RI
1	*α*-Pinene	C_10_H_16_	4.69	4.22	931
2	(+)-Sabinene	C_10_H_16_	2.54	4.93	943
3	*α*-Myrcene	C_10_H_16_	13.83	5.35	941
4	D-Limonene	C_10_H_16_	63.40	6.25	933
5	*α*-Ocimene	C_10_H_16_	0.87	6.74	914
6	*γ-*Terpinene	C_10_H_16_	0.21	6.98	913
7	*cis*-Linalool oxide	C_10_H_18_O_2_	0.82	7.22	858
8	Nonanal	C_9_H_18_O	0.23	7.93	920
9	3,7-Dimethylocta-1,6-dien-3-ol	C_10_H_18_O	0.92	8.01	939
10	(R)-(+)-Citronellal	C_10_H_18_O	0.32	9.33	928
11	α-Terpineol	C_10_H_18_O	0.56	10.53	928
12	Decanal	C_10_H_20_O	1.91	10.98	950
13	α-Citral	C_10_H_16_O	0.90	11.87	859
14	Geranyl acetate	C_12_H_20_O_2_	0.64	16.51	933
15	*α*-Copaene	C_15_H_24_	1.01	16.63	954
16	Germacrene D	C_15_H_24_	1.52	17.00	935
17	Caryophyllene	C_15_H_24_	2.60	17.82	937
18	α-Humulene	C_15_H_24_	0.35	18.80	927
19	Germacrene B	C_15_H_24_	0.46	20.03	910
20	α-Cadinene	C_15_H_24_	1.22	20.84	963
21	(1 S,4 S,7R,8 S)-1,4a-Dimethyl-7-(prop-1-en-2-yl) decahydronaphthalen-1-ol	C_15_H_26_O	0.23	24.24	940
22	Nootkatone	C_15_H_22_O	0.51	27.71	917
Monoterpenes hydrocarbon			85.54		
Oxygenated monoterpenes hydrocarbon			6.30		
Sesquiterpene hydrocarbon			7.16		
Oxygenated sesquiterpene hydrocarbon			0.74		
Total			**99.74%**		

RI: retention index relative to *n*-alkanes, RT: retention time (min), MF: molecular formula, : major compound, %: percentage.

Metabolomics and multivariate statistical analysis results revealed a good separation of the analyzed samples. The observed clustering patterns highlight the presence of group-specific metabolic fingerprints within the *Citrus* varieties studied. The clear separation of *C. aurantifolia* and *C. paradisi* suggests that these species possess unique biosynthetic pathways or environmental influences leading to divergent accumulation of volatile compounds. The overlap observed among *C. reticulata* varieties, *C. sinensis* varieties, and *C. limettioides* implies a more conserved metabolic profile, possibly reflecting closer genetic relationships or similar growth conditions. Nevertheless, the subtle differences captured by heatmap analysis indicate that even within these groups, minor chemical variations exist and may be explored for more refined classification.

## Conclusion

In this manuscript, we provide a comprehensive study of the volatile composition of the 12 most cultivated *Citrus* in Egypt. Forty-nine volatile organic compounds have been reported in all 12 varieties, most of them terpenoid (90–100%). Moreover, the essential oil components in *C. sinensis*, *C. reticulata* Blanco, *C. aurantifolia*, and *C. limettioides* cultivated in different regions corroborates some commonalities. Consequently, limonene, a hydrocarbon monoterpene, is invariably the most common ingredient in essential oils made from *Citrus* peels, making up typically between 60 and 95% of the oil. Also prevalent are the following substances: monoterpenes, which typically account for less than 30%, β-pinene, *α*-myrcene. Non-terpenoid or terpenoid compounds (aldehydes, ketones, and esters) are reported to be present (1–10%) or absent according to the cultivated region, but there are no commonalities among studies reporting these compounds to have an impact on the essential oil activity or not. Sesquiterpene hydrocarbons are the most varied group of all known chemicals, which generally account for the less content. On the other hand, multivariate chemometric approaches (PCA, PLS-DA, and sPLS-DA) revealed effectiveness in distinguishing between different *Citrus* groups based on their volatile organic compound profiles. The analysis revealed distinct clustering patterns that highlight biochemical variability among *C. reticulate* varieties, *C. sinensis* varieties, *C. limettioides*, *C. aurantifolia* and *C. paradise*. These findings highlight the potential of GC-MS-based chemometric analysis for the exploration of citrus diversity and could support future applications in quality control, and *Citrus*-derived product development. However, further validation using larger and geographically diverse sample sets is required before definitive biomarkers can be established.

## Supplementary Information

Below is the link to the electronic supplementary material.

Supplementary Material 1

## Data Availability

All datasets analyzed or generated during the study are included in the manuscript.
