# Supplementary material for: Volatile composition of essential oils from major Egyptian Citrus cultivars: a multivariate chemometric approach
Source: Sci Rep. 2026 Jul 6;16:20746. doi: 10.1038/s41598-026-60409-w (PMC13338266; doi:10.1038/s41598-026-60409-w)
Supplement: Supplementary file 1 — Supplementary Material 1 [file 41598_2026_60409_MOESM1_ESM.docx]

Volatile Composition of Essential Oils from Major Egyptian *Citrus* Cultivars: A Multivariate Chemometric Approach

Abeer H. Elmaidomy^1^, Esraa M. Mohamed^2^, Hebatallah S. Bahr^3^, Usama Ramadan Abdelmohsen^4*^

^1^Department of Pharmacognosy, Faculty of Pharmacy, Beni-Suef University, Beni-Suef 62514, Egypt. Abeer011150@pharm.bsu.edu.eg

^2^Department of Pharmacognosy, Faculty of Pharmacy, MUST, Giza, 12566, Egypt

^3^Department of Pharmacognosy, Faculty of Pharmacy, Nahda University, Beni-Suef 62513, Egypt. Hebatallah.samir@nub.edu.eg

^4^Department of Pharmacognosy, Faculty of Pharmacy, Deraya University, Minia 61111, Egypt.

Correspondence author: usama.ramadan@mu.edu.eg (U.R.A.).


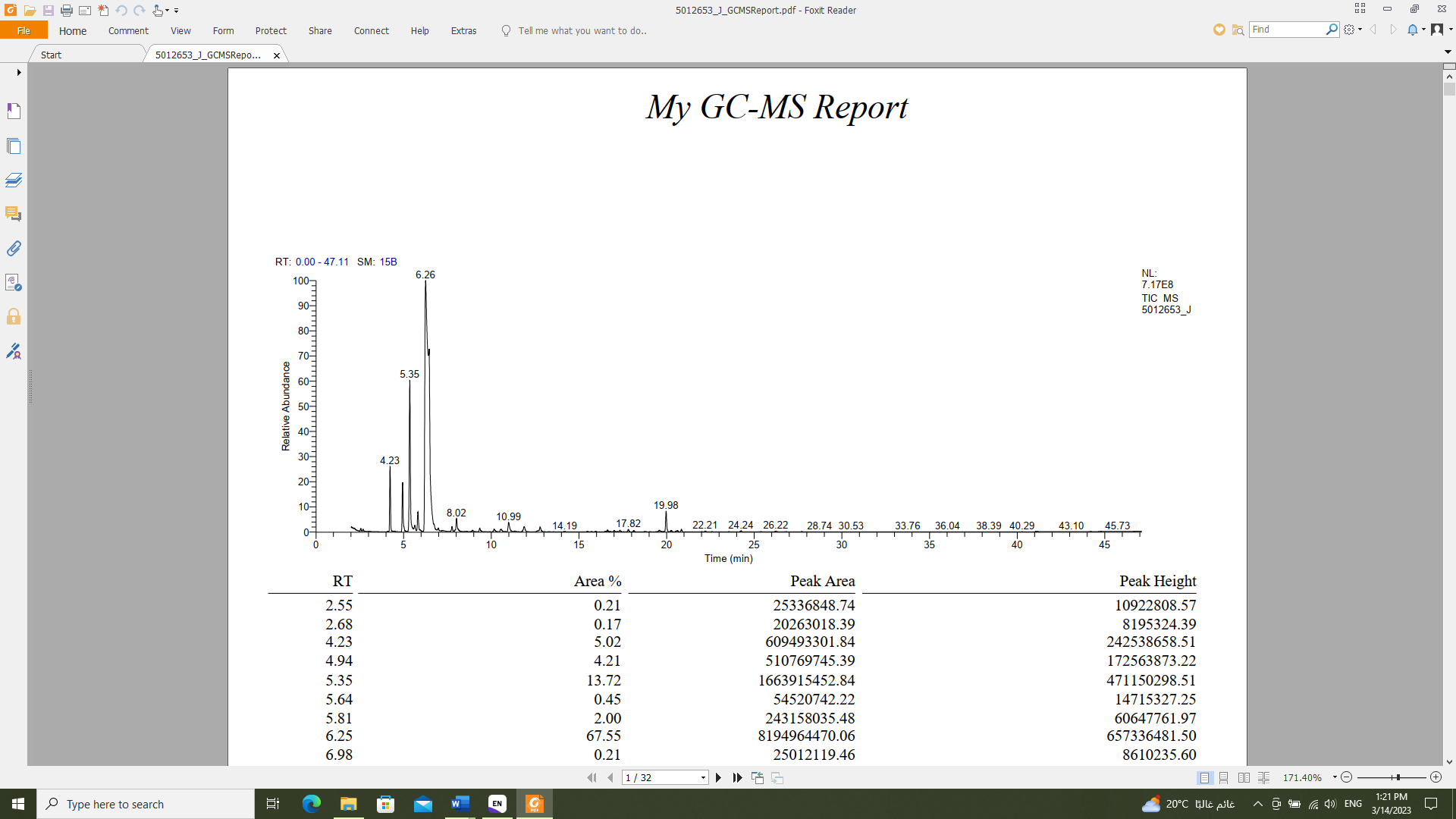


**A**


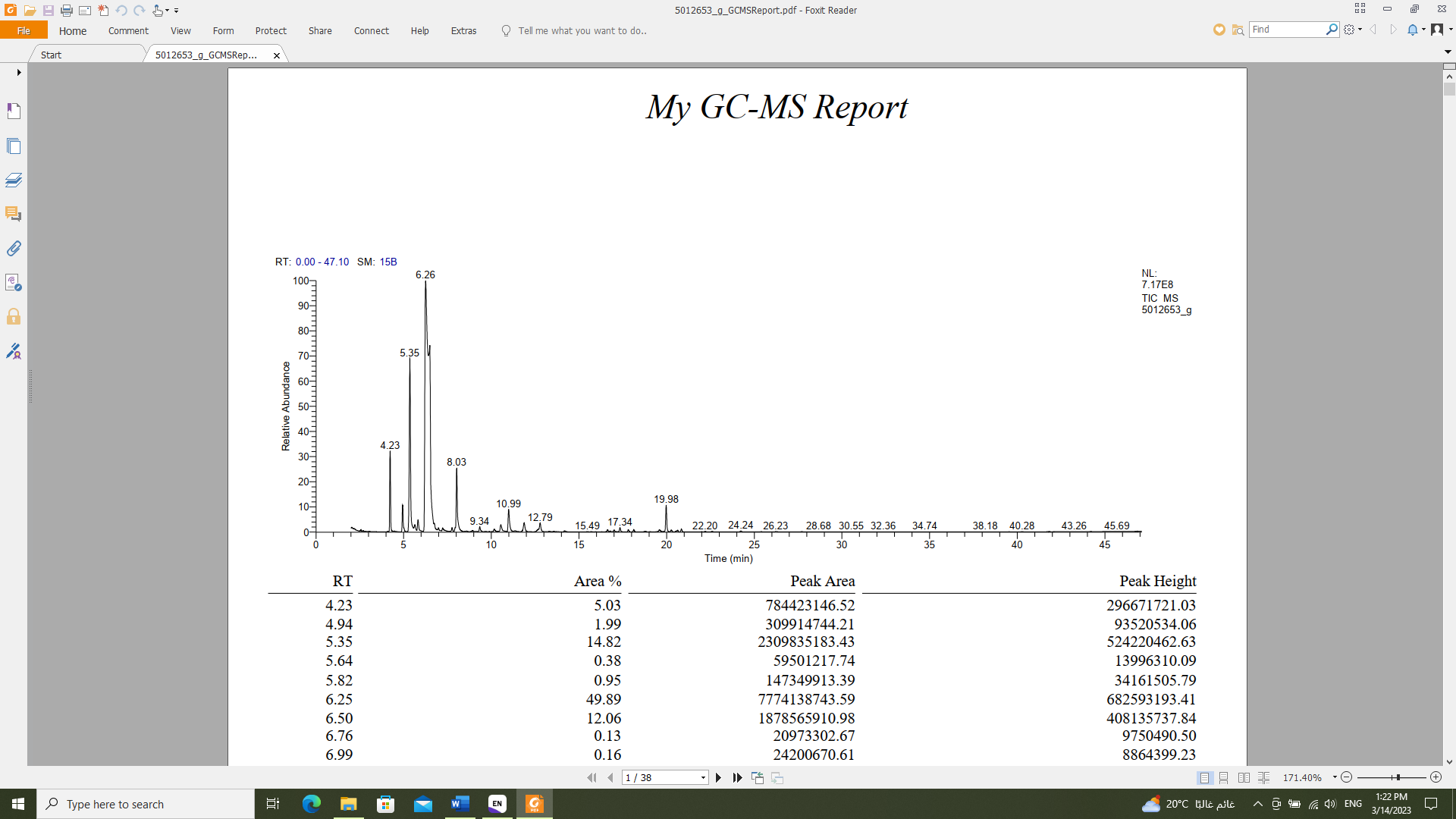


**B**


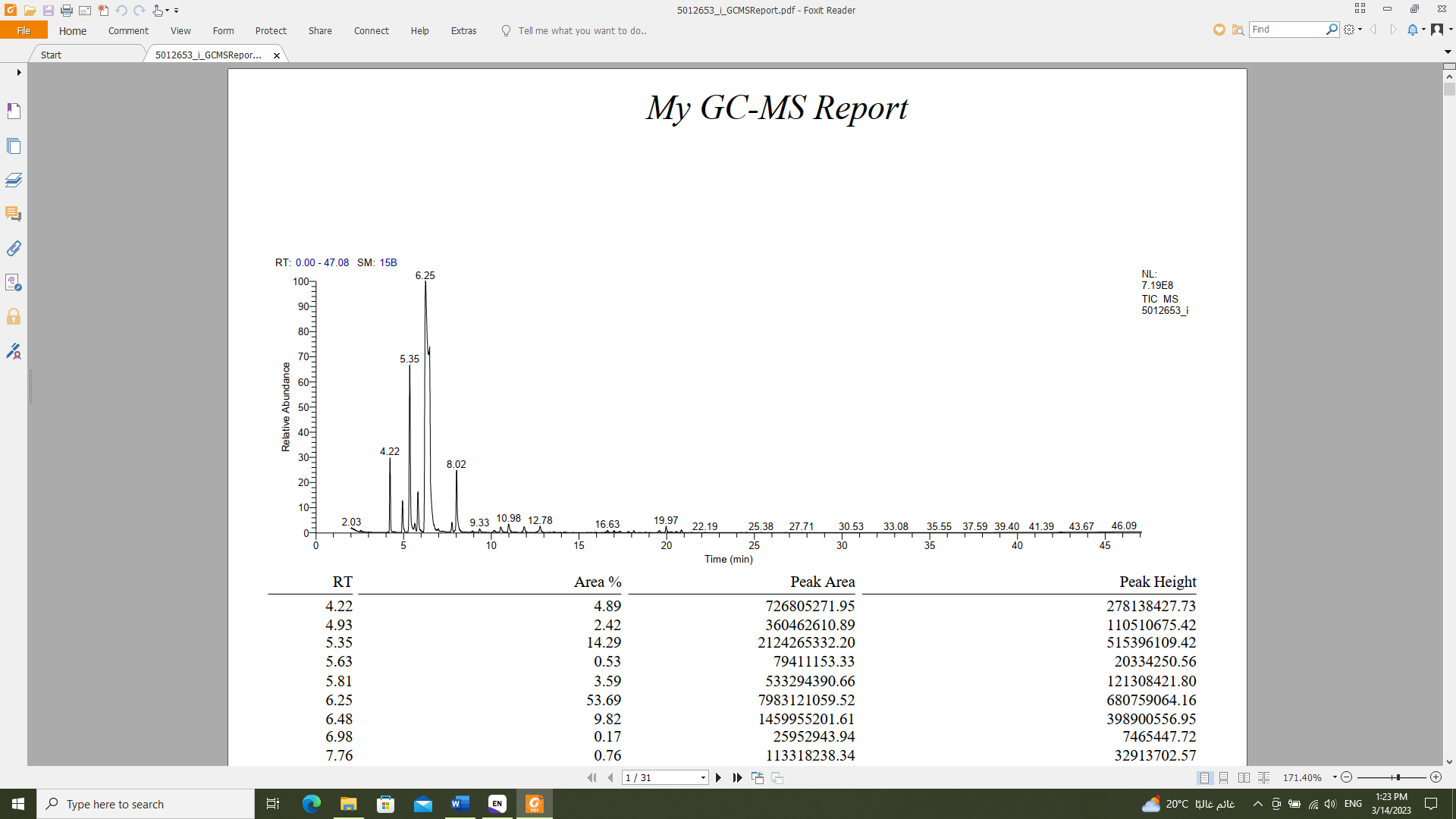


**C**


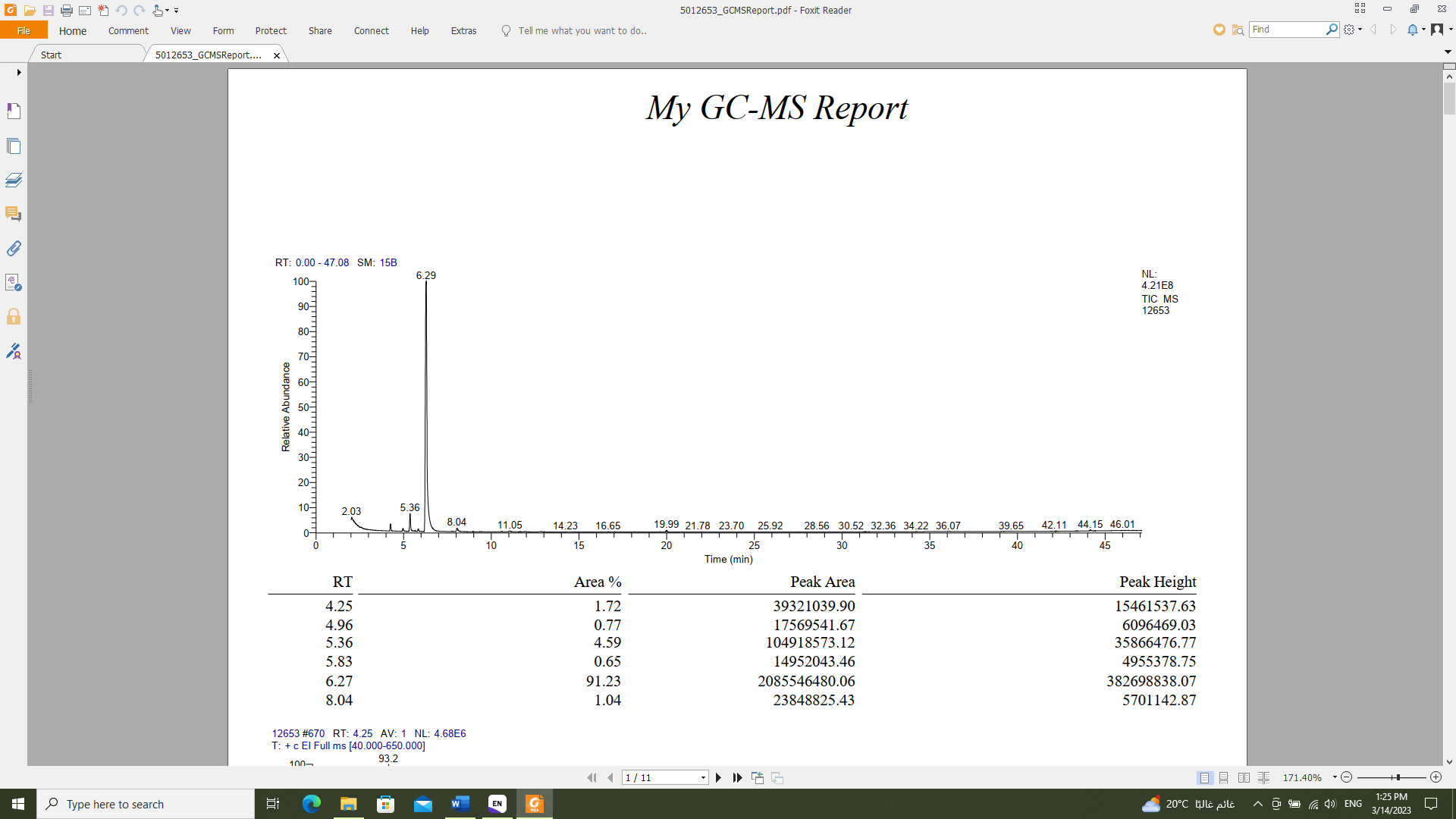


**D**

**Figure S1.** GC/MS spectrums for *Citrus sinensis* varieties: “Washington Navel (Navelina)” A, “Balady Seeded Orange” B, “Blood Orange” C, and “Sweet Orange Sukkari” D, peel oils.

**Figure S2.** Structures of identified compounds, using GC/MS analysis, from *Citrus sinensis* varieties: “Washington Navel (Navelina)”, “Balady Seeded Orange”, “Blood Orange”, and “Sweet Orange Sukkari”, oils isolated from peels.


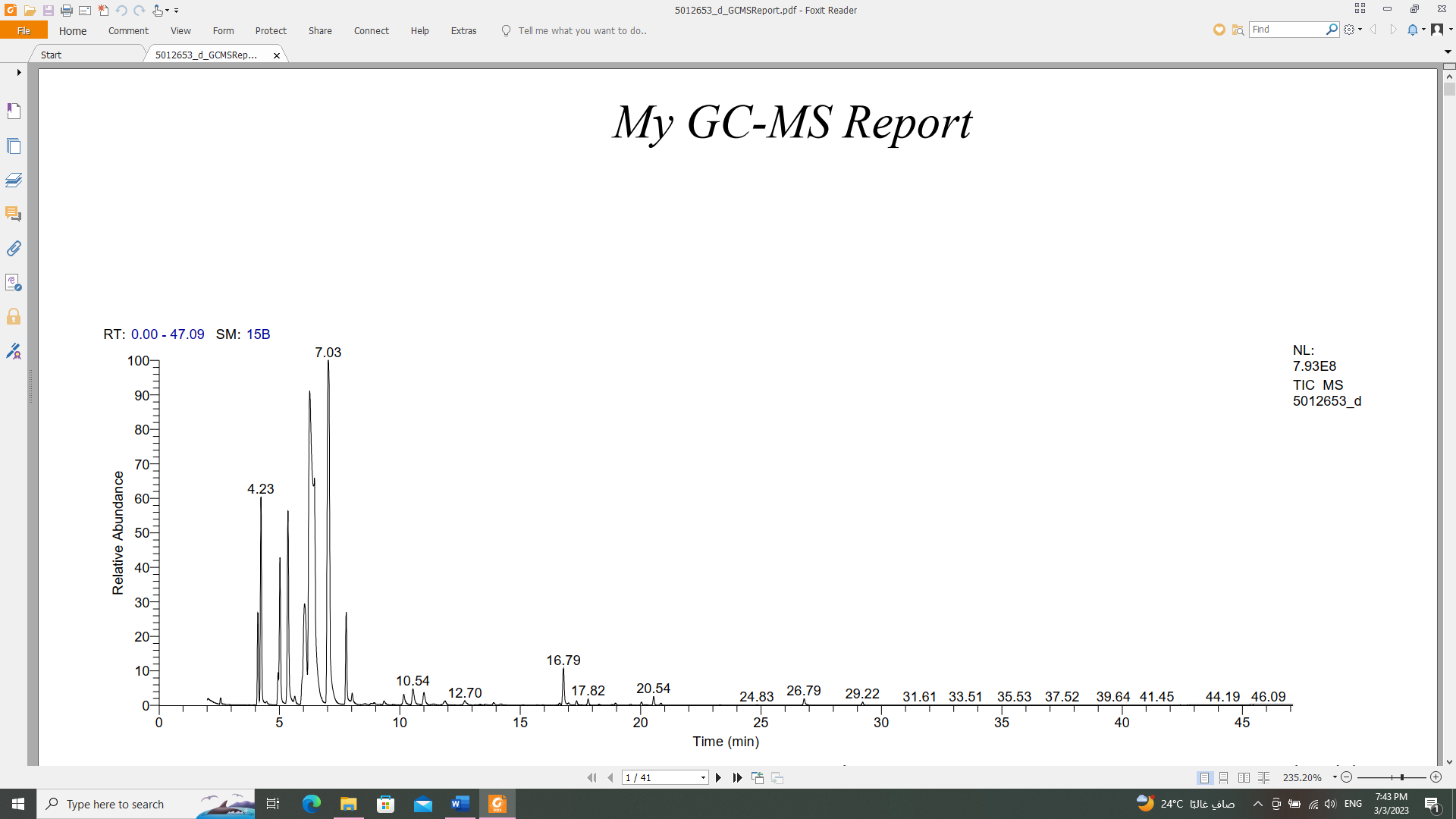


**A**


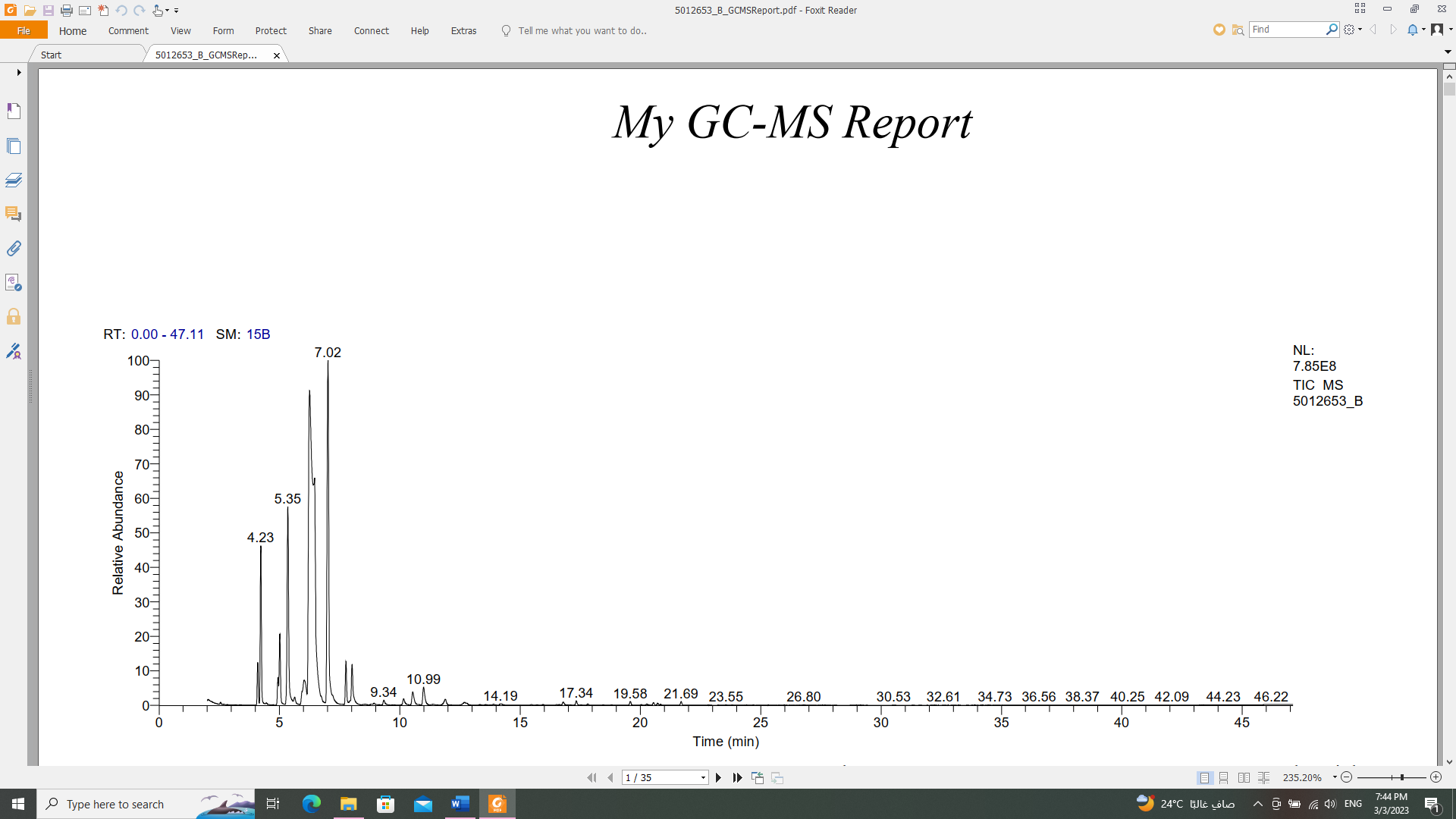


**B**


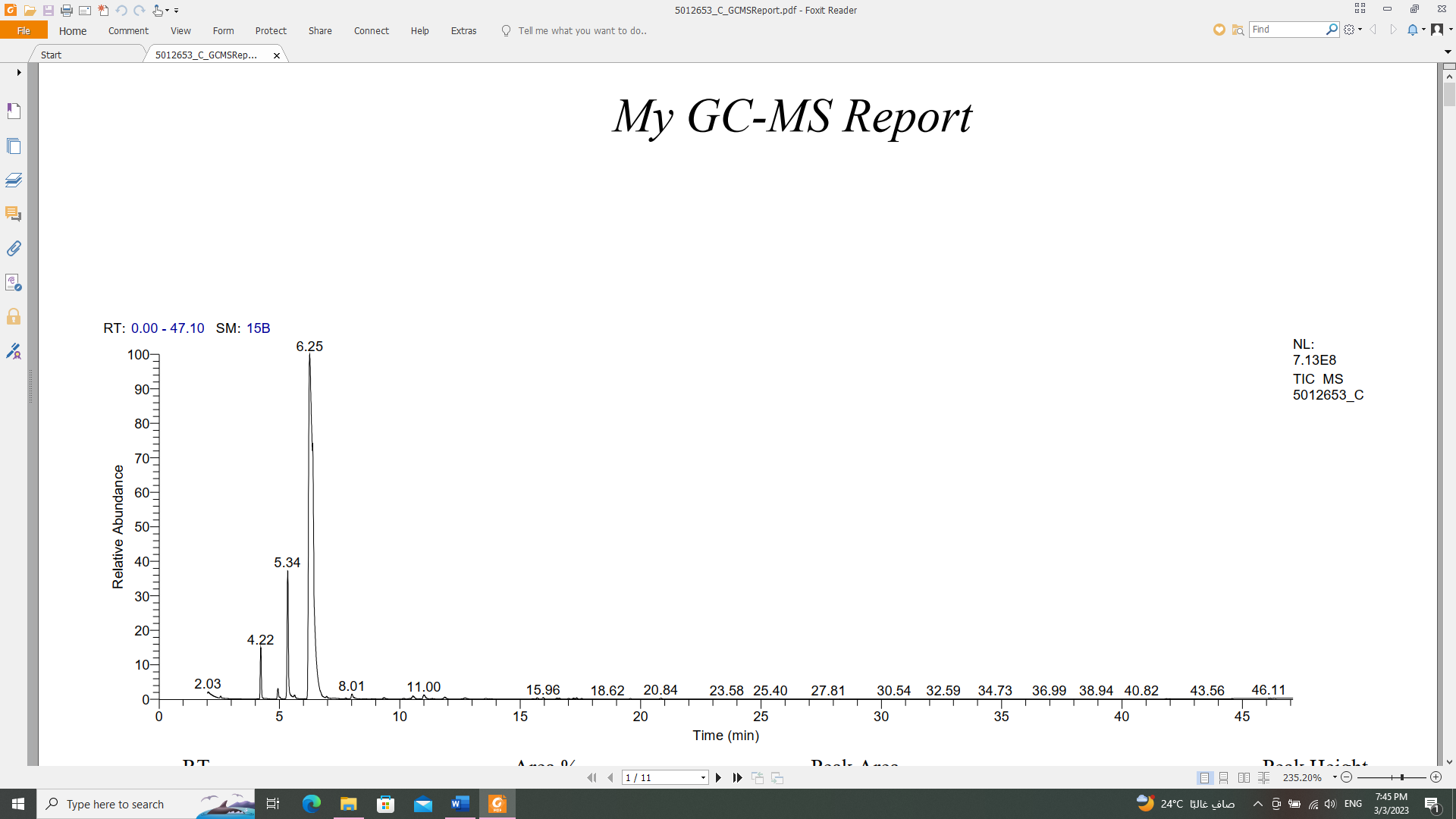


**C**


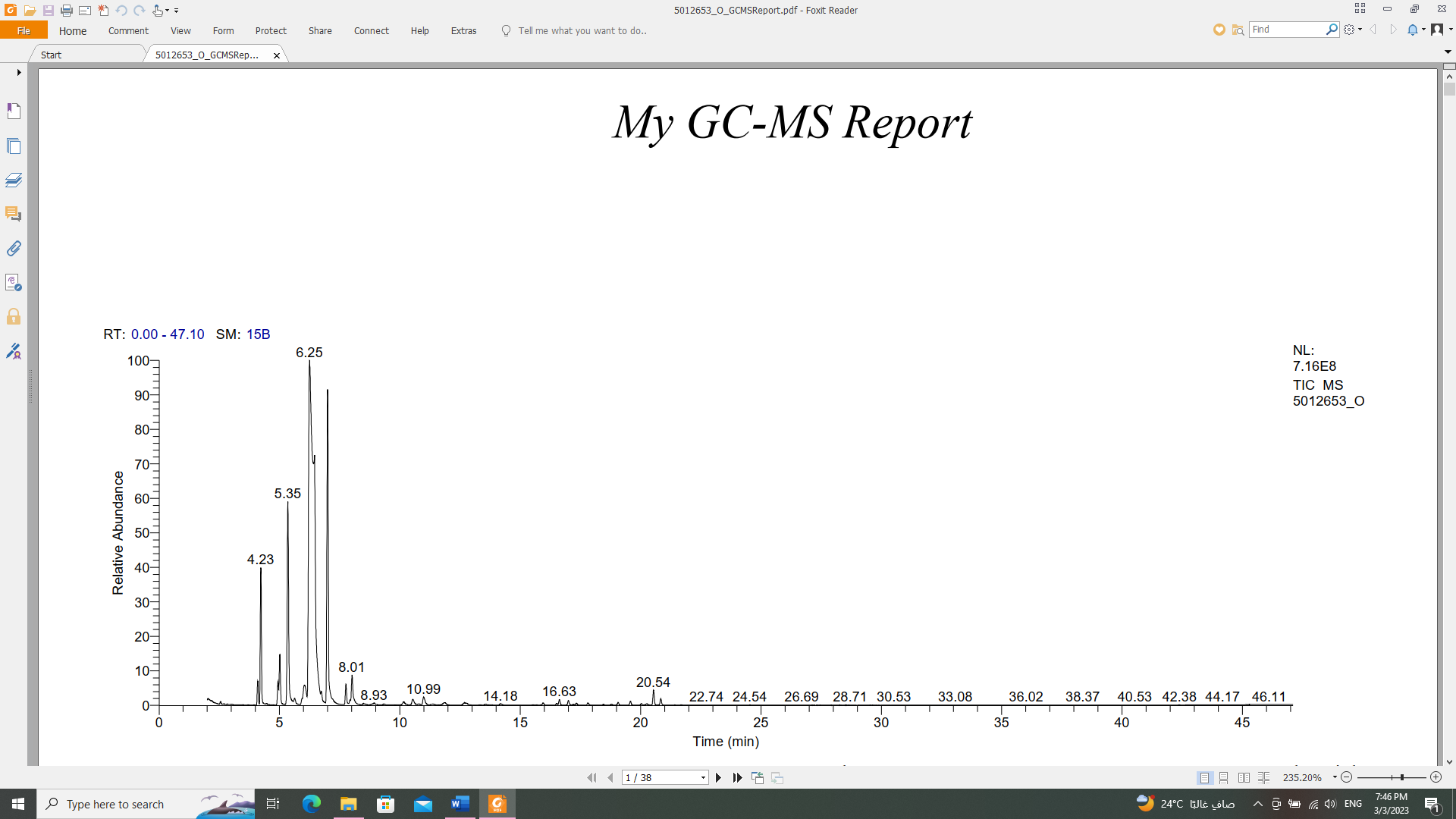


**D**


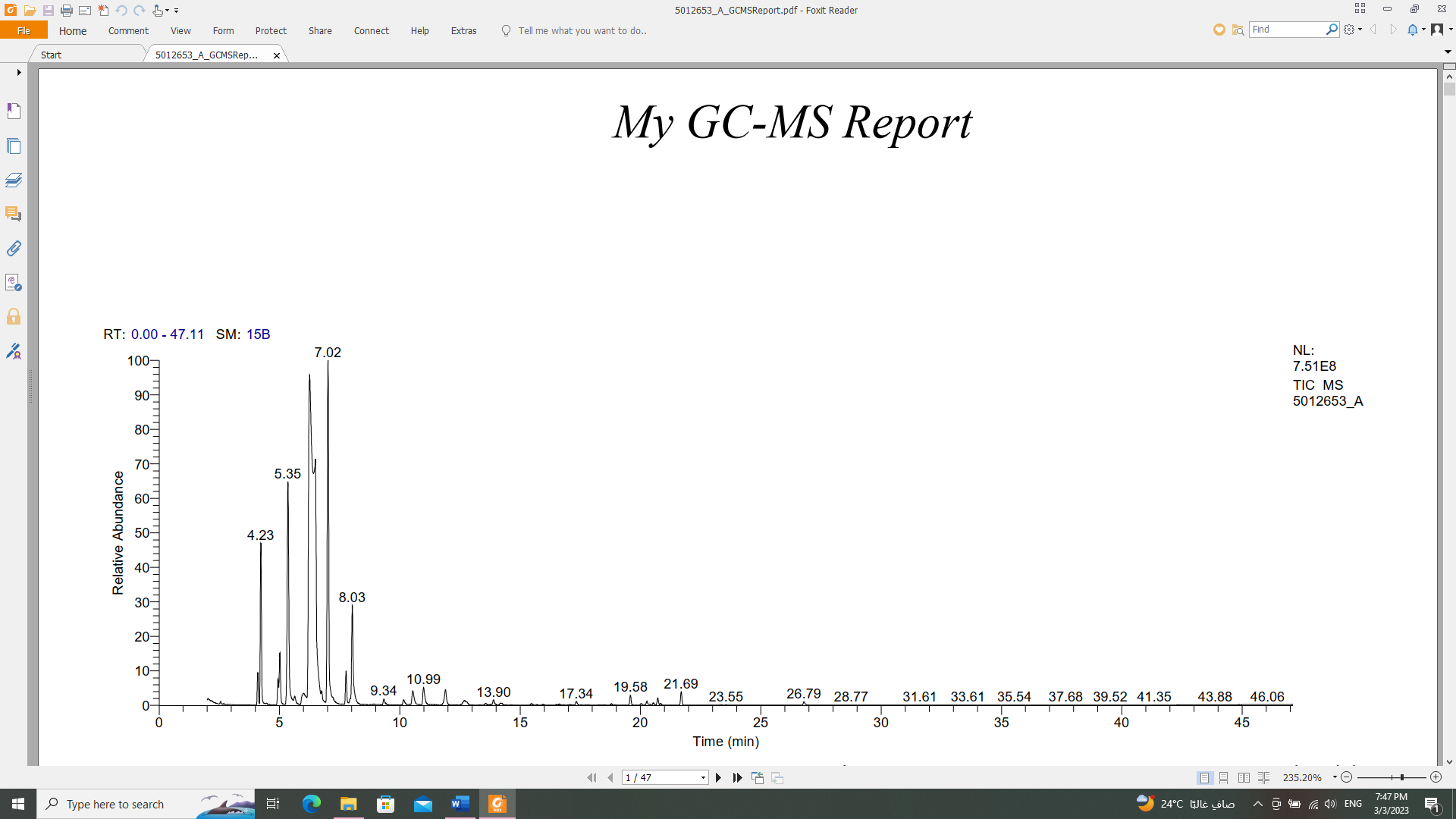


**E**

**Figure S3.** GC/MS spectrums for *Citrus reticulata* Blanco varieties: “Yousfy Balady” A, “Ponkan Chinese Honey Mandarin” B, “Murcott Mandarin” C, “Clementine Mandarin” D, and “Dancy Tangerine” **E,** peel oils.

**Figure S4.** Structures of identified compounds, using GC/MS analysis, from *Citrus reticulata* Blanco varieties: “Yousfy Balady”, “Ponkan Chinese Honey Mandarin”, “Murcott Mandarin”, “Clementine Mandarin”, and “Dancy Tangerine” oils isolated from peels.


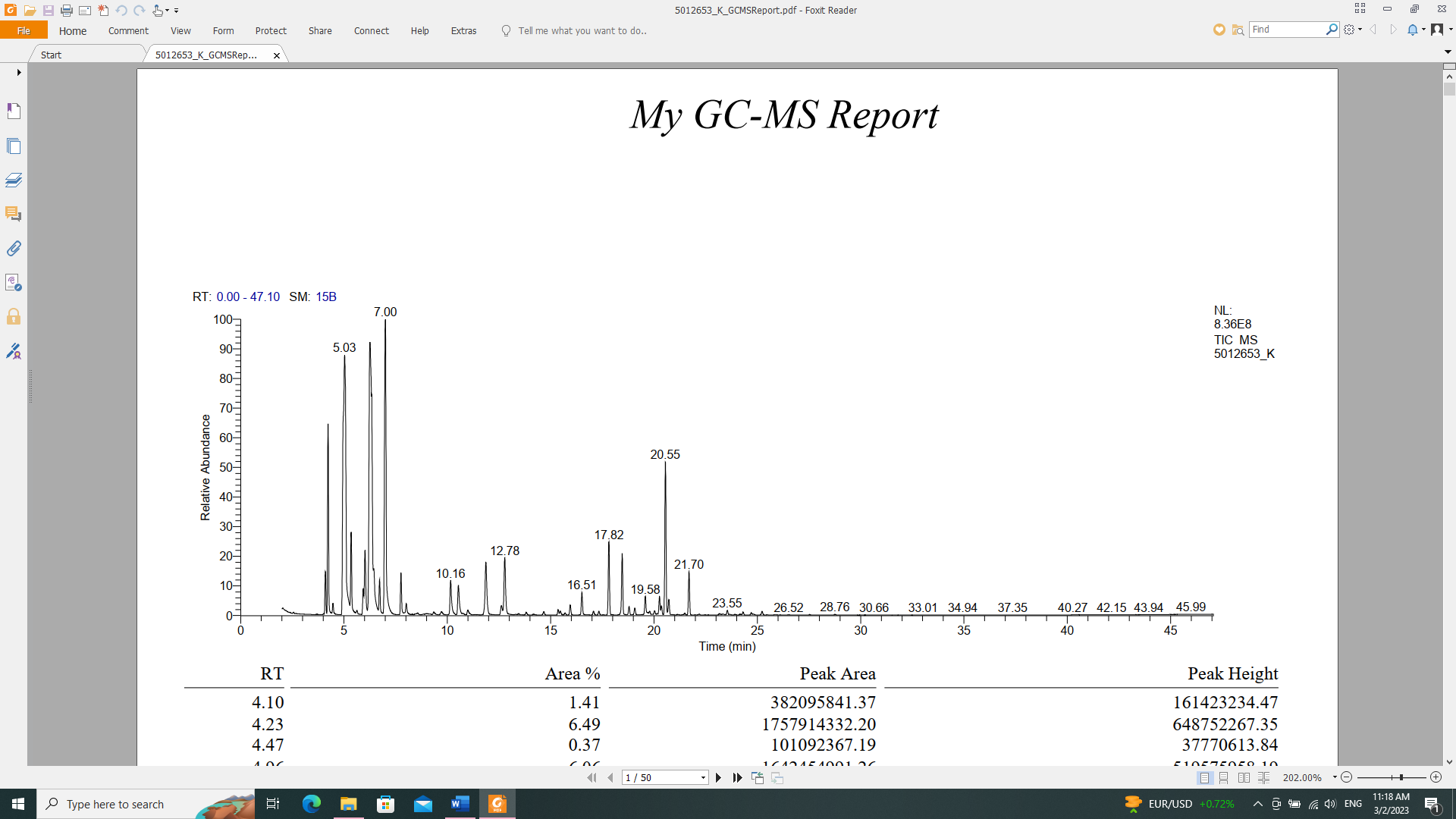


**A**


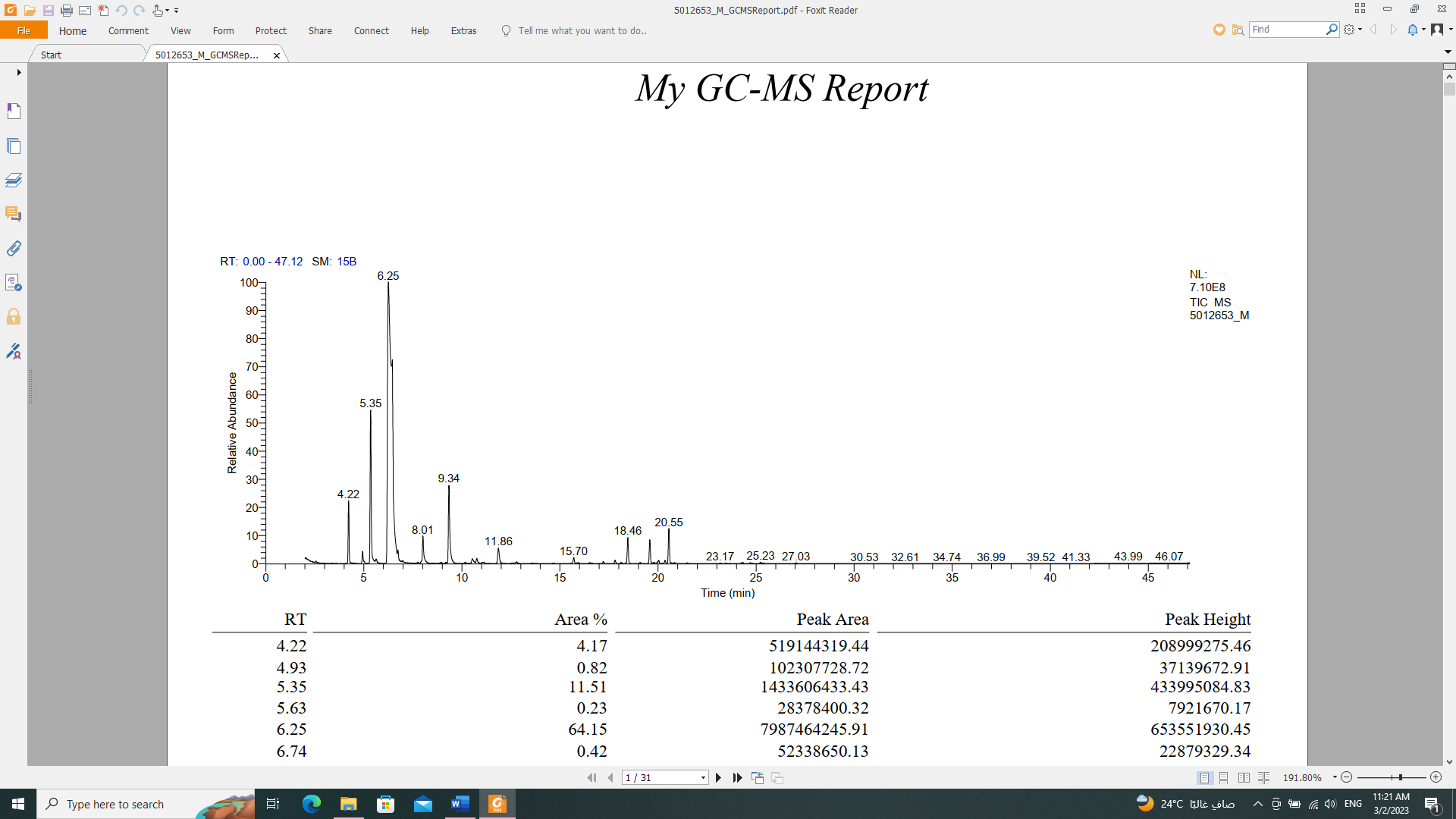


**B**

**Figure S5.** GC/MS spectrums for *C. aurantifolia* (A), and *C. limettioides* (B) peel oils.

**Figure S6.** Structures of identified compounds, using GC/MS analysis, from *C. aurantifolia,* and *C. limettioides* oils isolated from peels.


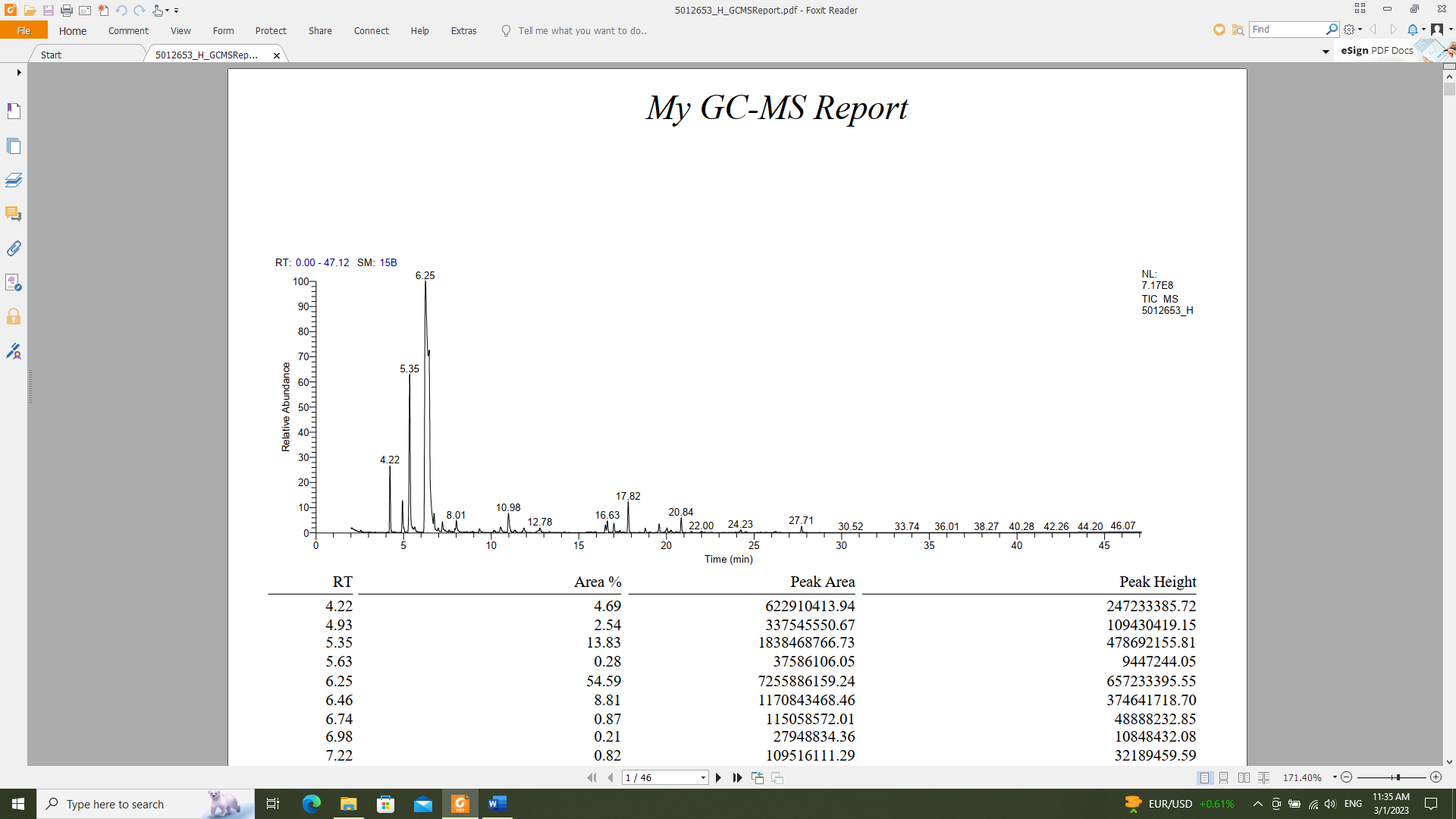


**Figure S7.** GC/MS spectrum for *Citrus paradisi* Macf. peel oil.

**Figure S8.** Structures of identified compounds, using GC/MS analysis, from *Citrus paradisi* Macf. oil isolated from peels.
